# Hydrolysis of Hemicellulose and Derivatives—A Review of Recent Advances in the Production of Furfural

**DOI:** 10.3389/fchem.2018.00146

**Published:** 2018-05-08

**Authors:** Frederic Delbecq, Yantao Wang, Anitha Muralidhara, Karim El Ouardi, Guy Marlair, Christophe Len

**Affiliations:** ^1^Ecole Superieure de Chimie Organique et Minerale, Compiègne, France; ^2^Sorbonne Universités, Universite de Technologie de Compiegne, Compiègne, France; ^3^Institut National de l'Environnement Industriel et des Risques, Verneuil-en-Halatte, France; ^4^Avantium Chemicals, Amsterdam, Netherlands; ^5^Materials Science and Nano-Engineering Department, Mohammed VI Polytechnic University, Ben Guerir, Morocco; ^6^Institut de Recherche de Chimie Paris, PSL University, Chimie ParisTech, Paris, France

**Keywords:** furfural, catalysis, green chemistry/safety biorefinery, biomass, dehydration, mechanism

## Abstract

Biobased production of furfural has been known for decades. Nevertheless, bioeconomy and circular economy concepts is much more recent and has motivated a regain of interest of dedicated research to improve production modes and expand potential uses. Accordingly, this review paper aims essentially at outlining recent breakthroughs obtained in the field of furfural production from sugars and polysaccharides feedstocks. The review discusses advances obtained in major production pathways recently explored splitting in the following categories: (i) non-catalytic routes like use of critical solvents or hot water pretreatment, (ii) use of various homogeneous catalysts like mineral or organic acids, metal salts or ionic liquids, (iii) feedstock dehydration making use of various solid acid catalysts; (iv) feedstock dehydration making use of supported catalysts, (v) other heterogeneous catalytic routes. The paper also briefly overviews current understanding of furfural chemical synthesis and its underpinning mechanism as well as safety issues pertaining to the substance. Eventually, some remaining research topics are put in perspective for further optimization of biobased furfural production.

## Introduction

**Scheme 1:**
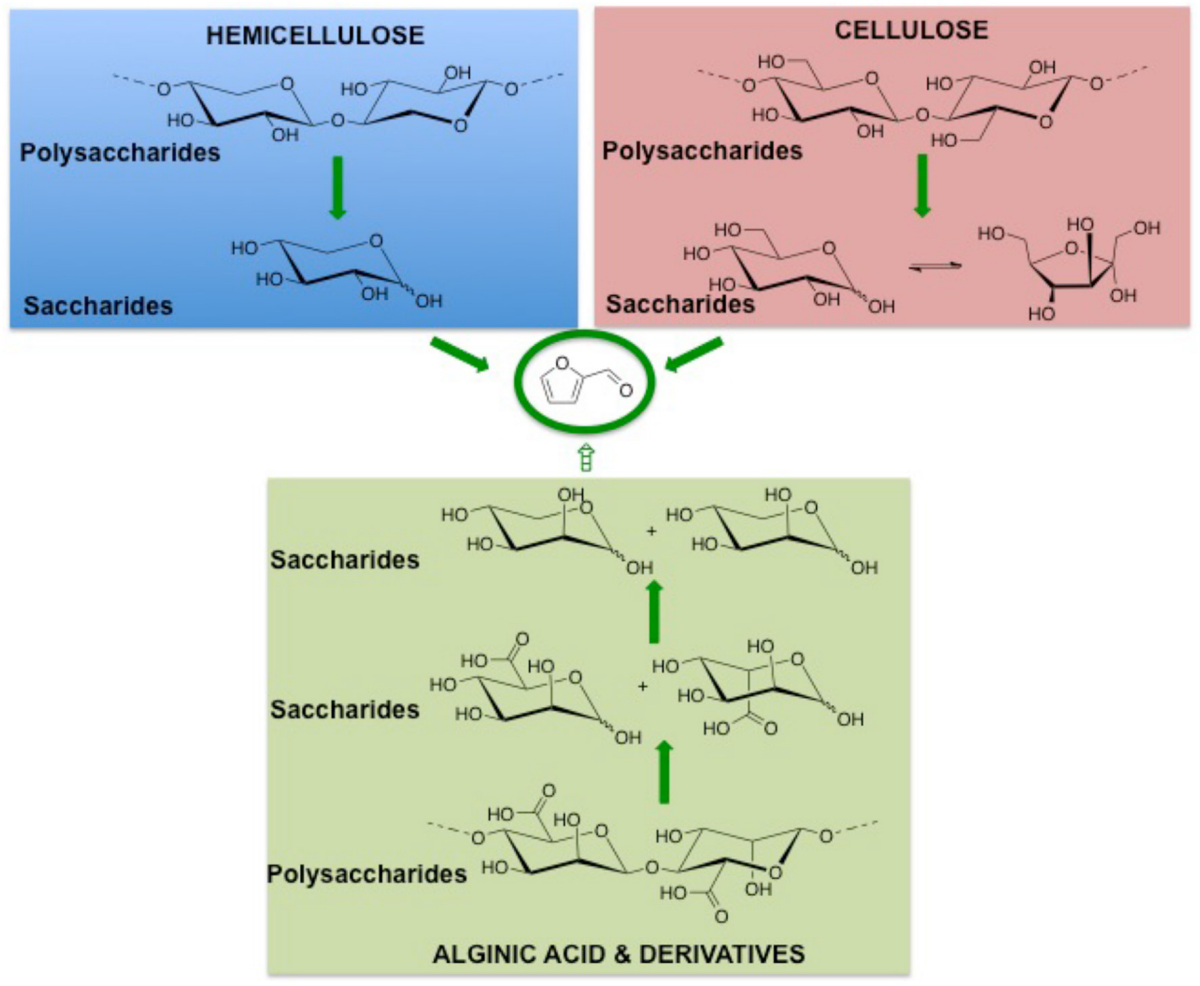
Sources of sugars for the integrated production of furfural.

**Scheme 2:**
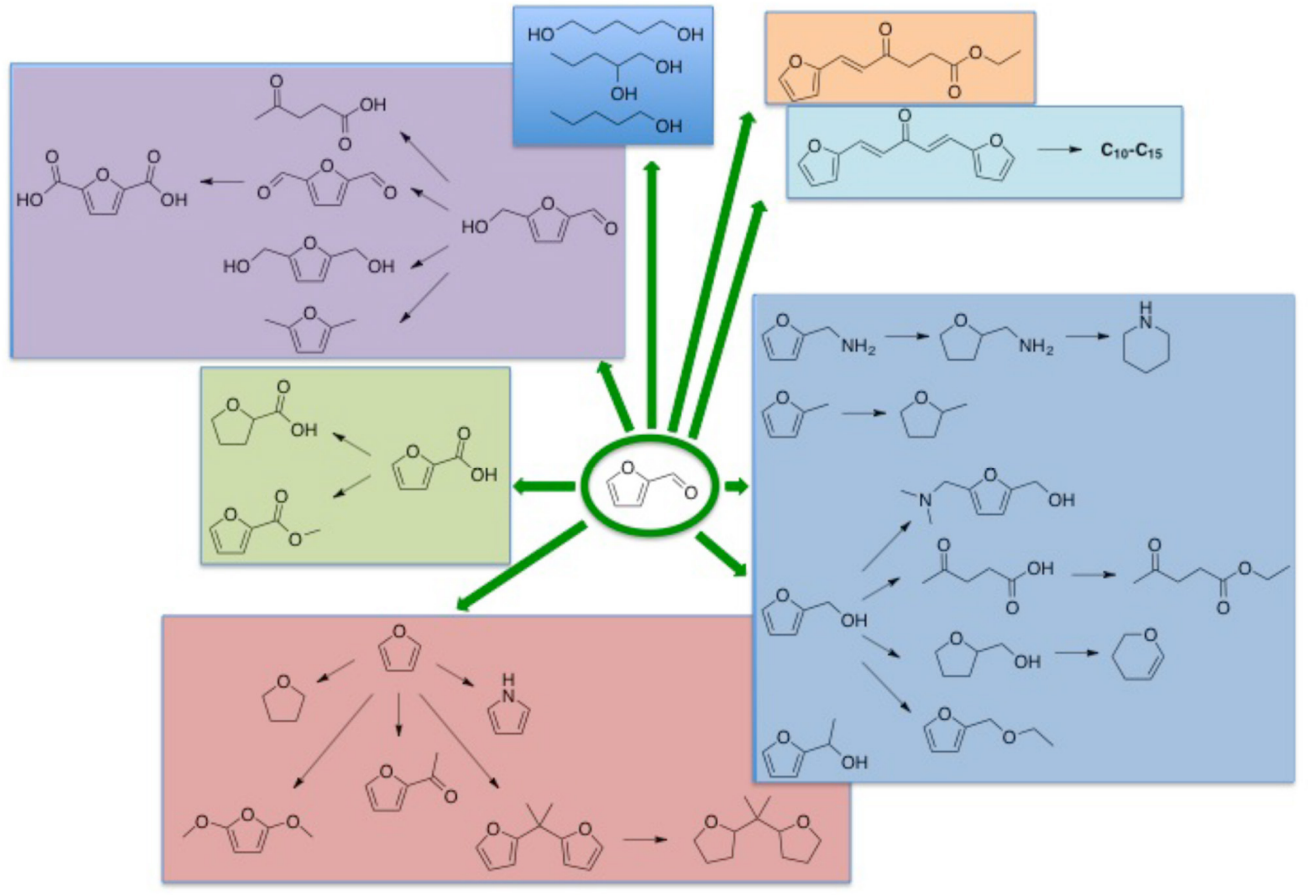
Representative synthesis of chemicals and fuel additives from furfural.

Nowadays, from a global point of view, the need for basic compounds continues to grow day by day. Our planet is confronted to the reduction of fossil resources, majoring the price of fossil-fuel based materials and the increase of greenhouse gas emissions (Harmaajärvi et al., 2004; Kamm et al., 2006; Brown and Brown, 2013) as well as increasing societal demand for industrial ecology aiming at disconnecting growth of production of goods and wealths from environmental impacts of all sorts and associated concepts (Jelinsky et al., 1992; Bourg and Erkman, 2003). These concerns require the intensive production of biorenewable resources including related residues through intensive processes into identified useful chemicals. The use of plant wastes as recycled feedstocks is one of the alternatives for minimizing the dependence on fossil oil (Hall, 1981; Hacker et al., 2009; Aresta et al., 2012). Chemical companies convert the renewable bioresources into biofuels, in platforms molecules for fine chemicals, agro-chemicals, and specialty chemicals such as bio-lubricants, natural fibers and bio-based solvents (Schieb et al., 2015). Several building blocks derived from renewable resources such as ethanol, glycerol, lactic acid, succinic acid, levulinic acid, are already in use or considered with potential importance in the near future (Sorensen et al., 2009). Among them, furanic compounds, such as 2-furaldehyde (furfural) and 5-hydroxymethylfurfural (HMF) having various industrial applications (Karinen et al., 2011; Lange et al., 2012) are conventionally produced from plant wastes feedstocks *via* several steps that led to relatively lower overall yield (Scheme 1). However, many reports have shown that one-pot and efficient production of furans could be achieved from biomass by well-optimized catalysts, solvents, equipment and process technology upgrades. Generally, furfural was derived from hemicellulose or other pentose rich polysaccharides classified in four main groups: xylans, mannans, xyloglucans, and β-glucans (Ebringerová, 2005). Recent researches have shown that other kinds of carbohydrates as alginate derivatives could also be exploited as feedstocks for furfural production (Scheme 1). As a molecule platform chemical, furfural permits to produce a large range of chemicals having different properties and utilities as solvents, plastics, fuel additives (Scheme 2). The purpose of the present article is to summarize the state of art in the field of furfural production from sugars and polysaccharides using homogeneous and heterogeneous strategies.

## Synthesis of furfural from sugars and polysaccharides without catalysts

### Dehydration in critical solvents

**Figure 1:**
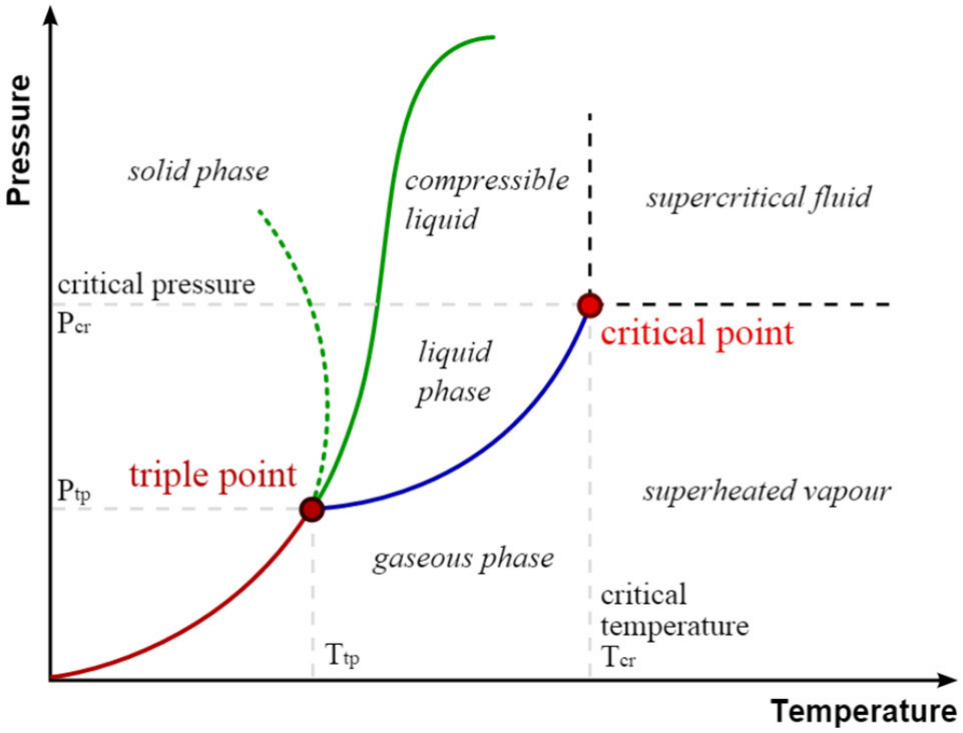
Critical point in a pressure-temperature phase diagram.

Critical solvents are characterized by a critical point which is obtained at specific pressure and temperature (Figure 1). When temperature and pressure of a fluid rise higher than relating values defining the critical point of the solvent, this fluid belongs to the family of supercritical solvents. When both: (i) the temperature and/or the pressure are lower than that of the critical point; and (ii) the temperature is higher than that of the boiling point with a pressure higher than 1 bar, subcritical solvent also called hot compressed solvent is obtained. Following physical characteristics: viscosity, density, static dielectric constant and, for the protic ones, ion dissociation constant are deeply modified by heating solvent under pressure, which give them unrivaled properties for some advanced applications in chemistry.

**Scheme 3:**
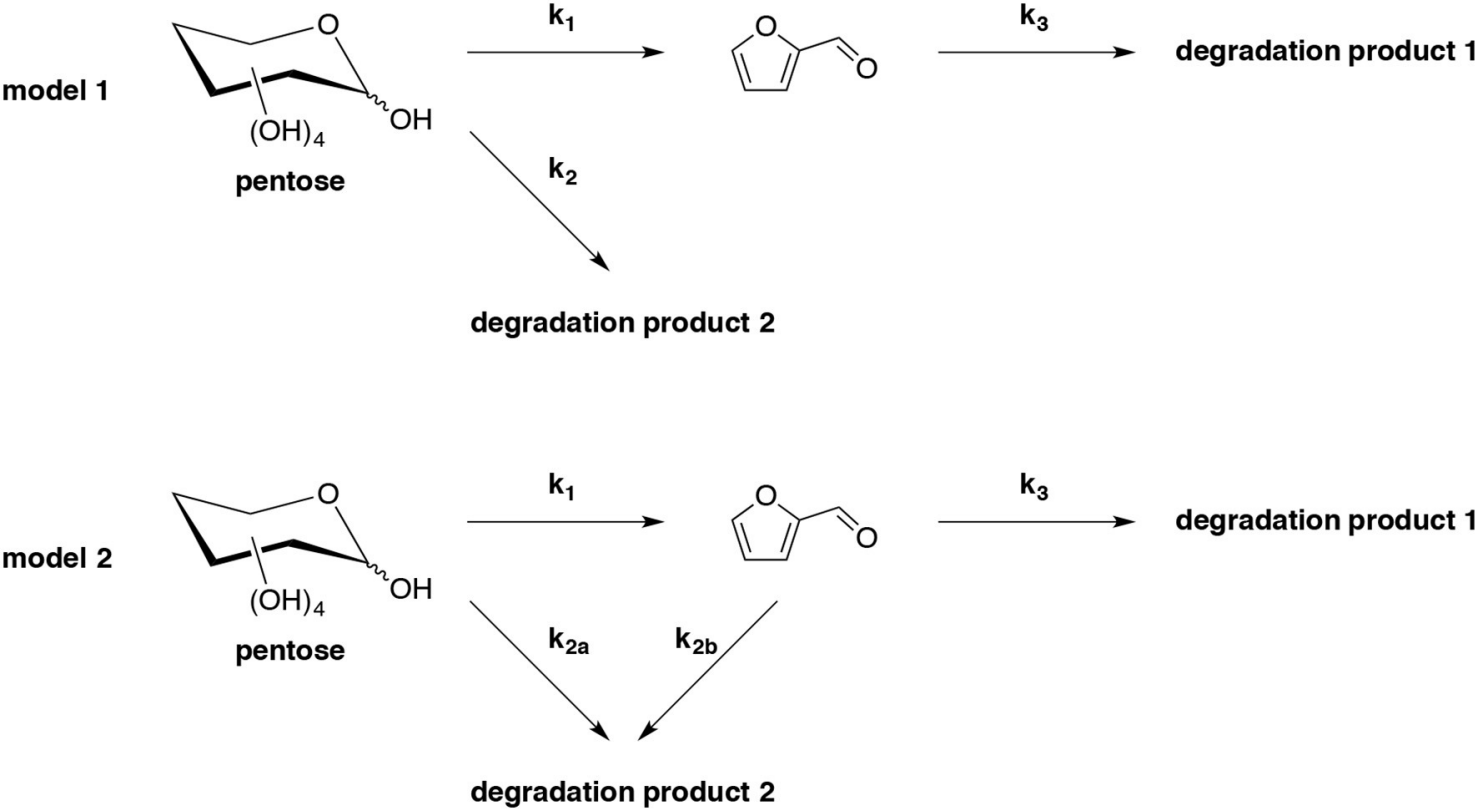
Kinetic models (model 1 and model 2) for pentose dehydration into furfural and the associated side-reactions in acidic or hydrothermal environment (Gairola and Smirnova, 2012).

Hydrothermal conversion of D-xylose and hemicelluloses to furfural by simultaneously furfural extraction with supercritical CO_2_ (sc-CO_2_) in catalyst-free conditions has been studied (Gairola and Smirnova, 2012). Two common reaction models of pentose dehydration (model 1 and model 2) were chosen and compared in terms of their ability to predict the influence of sc-CO_2_ extraction on furfural yield (Scheme 3). By optimizing the reaction temperature, time, pressure, CO_2_ flow rate and D-xylose concentration, a maximum furfural yield of 68% was furnished from 4% D-xylose initial loading at 230°C for 25 min at the pressure of 12 MPa and 3.6 g/min CO_2_ flow rate. Kinetics of D-xylose and L-arabinose dehydration demonstrated that the last model is more appropriate than the former one to explain the improvement of furfural yield by simultaneously extraction with supercritical CO_2_ during its formation. Additionally, the method was extended to native biomass as feedstock to produce furfural, it is clear that supercritical extraction improved furfural yield significantly. However, the best result derived from wheat straw furnished furfural in 29% yield, which was far lower than that from pure D-xylose. This result indicated the occurrence of side reactions with other hydrolysate components.

The use of another feedstock: sodium alginate and another solvent in presence or not of a catalyst permitted to have a fast decomposition of alginate at higher temperature (Jeon et al., 2015). However, in catalyst-free sub-critical water (pH = 7), the average molecular weight of products obtained at 250°C was higher than that for products generated under acid and base catalysts. These results suggested that catalyst-assisted hydrothermal treatment is favorable for the depolymerization of sodium alginate. Furthermore, only small amount of furfural formed without catalyst at 200 and 250°C, this phenomenon could be explained by the composition of alginate, which is mainly constituted of two monomeric subunits containing the carboxylic groups. These subunits are respectively D-mannuronic acid and L-guluronic acid, which are both hexuronic acids. Therefore, a decarboxylation processing step is necessary to realize the production of furfural. Obviously, the goal seems to be roughly achieved in subcritical water without catalyst.

### Dehydration using hot water pretreatment

Production furfural assisted by hot water pretreatment does not use in principle acid catalysts (no voluntary addition). Nevertheless some acids can be generated during the process. A lignocellulose liquid hot water pretreatment process was recently developed by direct recycling spent liquor (Lu et al., 2016). During hot water pretreatment, approximately 10.0 g of corn stover and 200 mL of distilled water at 180°C for 30 min furnished 8.0 g/L D-xylose and 0.3 g/L furfural and further increased to 21.3 g/L D-xylose and 4.2 g/L furfural after recycling of the spent liquid. The improvement of furfural yield was attributed to the fact that the formation of by-product acetic acid that will facilitate D-xylose dehydration to furfural, increased from 3.2 to 9.8 g/L as the number of recycling steps increased from 0 to 6. In catalyst-free conditions (without added acid), integration of biomass pretreatment with fast pyrolysis by hot water extraction (HWE) and electron beam (EB) irradiation showed that HWE could be used to reduce the formation of carboxylic acid and ketones whilst improving the yield of target organic compounds (Mante et al., 2014). Besides, EB irradiation has a good potential to increase the formation of useful furanic aldehydes (furfural and HMF) and decrease the yield of hydroxyacetaldehyde by-product.

The reaction kinetics in high-temperature water was studied for the production of furfural from D-xylose as monomer feedstock (Hua et al., 2016). The results indicated that high-temperature water has the potential to substitute solid and liquid acids as a catalyst since it efficiently promotes the transfer of furfural initially formed in the water phase into the organic phase. In this study, ethyl butyrate with its excellent distribution coefficient permitted to furnish furfural in 75% yield at 200°C for 3 h. This yield was superior to that yield obtained without extraction solvent (75 vs. 50%). In addition, the author also calculated the kinetic order of D-xylose dehydration to furfural in high-temperature water from 160 to 200°C. They found a kinetic order of the reaction of 0.5, associated dehydration rate constant K_0_ = 1.82.10^5^ (mol/dm^3^)^0.5^/min, and an activation energy (Ea) of 68.5 KJ/mol. In order to further investigate the conversion of D-xylose to furfural, furfural formation mechanism from D-xylose and solvent effects on its formation was investigated by density functional theory (DFT) (Wang et al., 2015). Kinetic and thermodynamic analyses proposed that D-xylulose could be the key intermediate that leads to the formation of furfural, and liquid water could stabilize both reactants and transition states with unsaturated C-C bonds, which is favorable to furfural production. This study also indicated a promising way to produce furfural from D-xylose by involving D-xylulose. Isomerization of D-xylose to its corresponding ketose isomer in high yield *via* a simultaneous-isomerization-and-reactive-extraction (SIRE) scheme was previously reported (Li B. et al., 2013). Concentrated and purified D-xylulose by back-extraction (BE) into an acid medium, and then rapidly dehydrated the D-xylulose sugar to furfural at relatively low temperature with no additional catalyst was studied. Furfural yield of 68% was achieved from D-xylulose at 110°C for 90 min, and was further improved to 90% with methyl isobutyl ketone (MIBK) as extract solvent or 85% in 5 min by replacing partial water with dimethyl sulfoxide (DMSO). The author also pointed out that the mild process conditions resulted in minimal chemical and energy inputs and have significant favorable impact on the overall process economics relative to D-xylose dehydration at 170°C, in spite of the additional unit operations involved. Besides, technical, economic and environmental performances for producing ethanol and furfural from *Pinus patula* bark, on a biorefinery concept was developed (Moncada et al., 2016). Based on different levels of heat integration, three scenarios were evaluated, the results showed that fully integrated plus cogeneration scheme was superior to fully energy integrated and non-integrated scheme from both point view of production costs and environment.

**Scheme 4:**
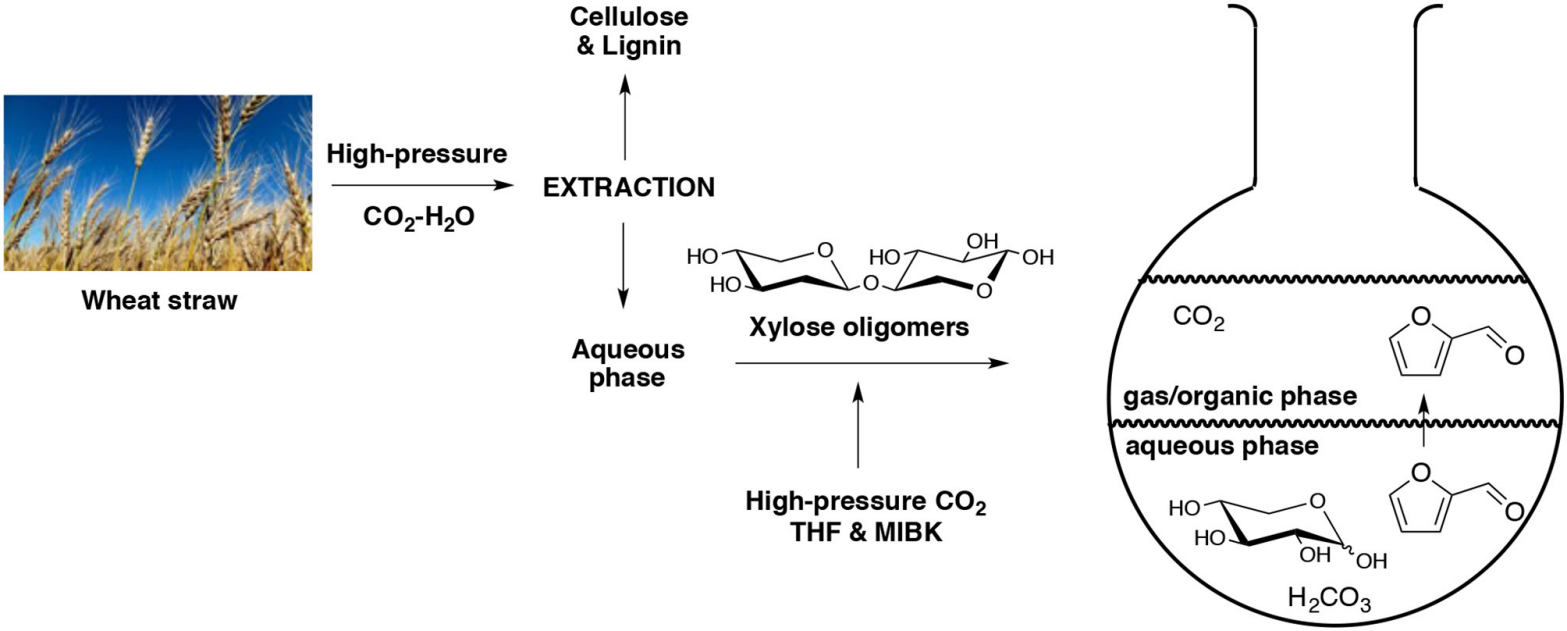
Production of furfural in an aqueous biphasic system using high pressure of CO_2_ as catalyst (Morais et al., 2016).

Simultaneous extraction-hydrolysis of lignocellulosic biomass by means of high pressurized CO_2_ and H_2_O was developed (Morais et al., 2016). In this work, the resulted water-soluble hydrolysate was also exposed to CO_2_ at higher pressure and temperature in presence of MIBK as the extractive solvent and tetrahydrofuran (THF) as co-solvent. At 200°C and 50 bar of CO_2_, the residual hemicellulose was decomposed into its pentose sub-units with 81% of overall conversion (Scheme 4). The optimized dehydration process required a temperature of 180°C and a residence time of 60 min giving a final furfural selectivity of 63%. In another paper, the same research team reported that they were able to produce furfural directly from D-xylose using a mixture of water and THF (Morais and Bogel-Lukasik, 2016). The dehydration was carried out under higher pressure of CO_2_ at 180°C for 1 h. They obtained, respectively 70 and 84% of furfural yield and selectivity.

## Synthesis of furfural from sugars and polysaccharides using homogeneous catalysts

Dehydration of D-xylose and derivatives was studied in presence of either mineral acids (H_2_SO_4_, HCl, H_3_PO_4_), organic acid (formic, methanesulfonic acid, maleic acid, succinic acid) or metal salts [FeCl_3_, AlCl_3_, CrCl_2_, CrCl_3_, CuCl_2_, NaHSO_4_, KAl(SO_4_)_2_, Al_2_(SO_4_)_3_] in water or in aqueous biphasic systems (ABS) using conventional thermal activation or microwave technology.

### Dehydration in presence of mineral acids

In many industrial furfural production processes, usual mineral acids, such as sulfuric acid, phosphoric acid, are generally used as catalysts. Nevertheless, other mineral acids have been studied in the last decade. A low acid hydrothermal (LAH) fractionation was developed to transform hemicelluloses isolated from *Giant Miscanthus* into D-xylose-rich hydrolyzate which is converted at 180°C in presence of H_2_SO_4_ into “furfural side-product” with 53% yield (Kim T. H. et al., 2016). Starting from a prehydrolyzate of aspen and maple chips, furfural was produced by use of H_2_SO_4_ as acid catalyst and heating the mixture in a range of temperature from 160 to 260°C (Mazar et al., 2017). With 3.6 kg/m^3^ of H_2_SO_4_, a value of 78% furfural yield was reached at 240°C. The authors noted that prior lignin removal from the pre-hydrolyzate did not bring any significant enhancement in the yield of furfural generated in this case.

Different additives such as thiourea, NaHSO_4_, NaCl were also tested as potential improvers of the dehydration process. For example, the impact of thiourea additive in the furfural production process from corncobs treatment using H_2_SO_4_ was described (Xu et al., 2017). They reported that thiourea had for effect to improve the furfural yield that reached 61% under a liquid-solid ratio of 2:1 instead 34% with only H_2_SO_4_. The transformation needed a temperature of 170°C and an acid concentration of 0.5 M. The authors explained that thiourea as an additive is supposedly acting as a blocking agent of the furfural resinification. In the same period, addition of NaHSO_4_ as a catalyst promoter was reported for the direct production of furfural from bagasse (Yazdizadeh et al., 2016). The optimized condition was set up at 160°C under 8 bar. With 23% of NaHSO_4_ content, a maximum of 9% furfural yield was reached after 50 min of residence time. NaCl as additive generated Cl^−^ ions, which promote the formation of the 1,2-enediol from the acyclic form of D-xylose, and thus enhances the production of furfural in aqueous acidic solution at temperatures between 170 and 200°C (Marcotullio and De Jong, 2010). The addition of NaCl in H_2_SO_4_ led to good furfural selectivity (90%). Variety of chloride salts as additives were studied and revealed positive effects in terms of reduction of mineral acid needs for the conversion. All of them also showed advantageous effect on furfural yield and selectivity except for FeCl_3_; however, they obtained exceptionally high D-xylose reaction rates, which deserved more investigation.

In order to substitute water for the dehydration other eco-friendly solvents such as γ-valerolactone (GVL) were tested and quantification of the effects of polar aprotic organic solvents on the acid-catalyzed conversion of D-xylose into furfural was reported (Mellmer et al., 2014). The use of GVL instead of water decreased the activation energy barrier for D-xylose dehydration from 145 KJ/mol to 114 KJ/mol, whereas the same parameter for furfural degradation increased from 85 KJ/mol to 105 KJ/mol. Accordingly, furfural selectivity from D-xylose of up to 75% could be achieved in GVL using H_2_SO_4_ as the catalyst, compared to only 50% furfural selectivity from D-xylose in H_2_O. The polar aprotic solvents have an influence on the stabilization of the acidic proton relative to the protonated transition states, which resulted in accelerated reaction rates for these acid-catalyzed biomass conversion reactions. The author suggested that the proton of strong solid Brønsted acid catalysts, such as H-beta, become solvated during the liquid-phase catalytic reactions, while the conjugate bases of the associated strong Brønsted acid catalysts have little effect on proton reactivity.

Among the novel alternative technologies developed in chemistry and chemical engineering, microwave heating was applied to produce furfural from D-xylose (Vaz Jr and Donate, 2015). The highest furfural yield of 64% was observed at 200°C for 10 min at aqueous HCl concentration (4 mg/mL), simultaneously with 95% of D-xylose conversion. The conversion of D-xylose and xylan to furfural by microwave-assisted reaction in HCl aqueous media was reported at 180°C for 20 min with a solid-liquid ratio of 1:100 and a pH adjusted to 1.12 with a solution of HCl (0.1 M). This optimized conditions furnished furfural in 38 and 34% yields, respectively from D-xylose and xylan (Yemiş and Mazza, 2011). However, furfural yield obtained from wheat straw, triticale straw and flax shives were 48, 46, and 72%, respectively. Under the optimized conditions, HCl is the most effective catalyst for furfural production from D-xylose and xylan compared to H_2_SO_4_, HNO_3_, H_3_PO_4_, CH_3_COOH and HCOOH. Hydrothermal transformation of giant reed (*Arundo donax* L.) to furfural and levulinic acid was described under microwave irradiation in the presence of diluted HCl (Antonetti et al., 2015). Furfural and levulinic acid theoretical yield of up to 70 and 90% were achieved under optimized conditions: biomass (0.35 g), water (5 g) with HCl (1.68 wt%) and 210°C for 15 min. It was also demonstrated that microwaves were shown to represent a very efficient alternative to the traditional heating route to give furfural and levulinic acid. However, when water was adopted as reaction medium, condensation by-products named humins was often observed especially at high reaction temperature also under microwave irradiation.

Recently, the additions of organic solvents such as MIBK, THF, toluene, cyclopentyl methyl ether (CPME) which can isolate the furfural formed from aqueous phase and further inhibit the occurrence of side-reactions have been demonstrated as an efficient method to improve furfural yield. Catalytic performance of heteropolyacids (HPAs) in the dehydration of D-xylose to furfural in different solvent system (DMSO, water, water-toluene or water-MIBK) was studied (Dias et al., 2005a). In this work, H_3_PW_12_O_40_ showed higher selectivity to furfural (64–69%) in comparison with H_4_SiW_12_O_40_ (52–64%) and H_3_PMo_12_O_40_ (inferior than 27%) in DMSO. However, H_4_SiW_12_O_40_ is the most effective one at 140°C for 24 h in water-MIBK biphasic system with furfural yield of 51%. For H_3_PW_12_O_40_ and H_4_SiW_12_O_40_ selectivity toward furfural production is higher for toluene-water than for DMSO for conversions up to 80%. In this work, water-MIBK did not seem to be a good solvent system to produce furfural compared to water-toluene or DMSO with tungsten-containing catalytic system.

**Scheme 5:**
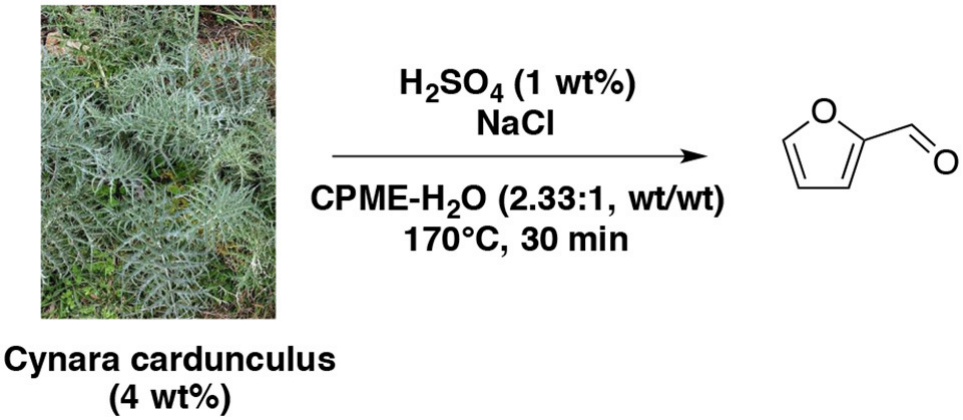
Production of furfural from cardoon in the presence of H_2_SO_4_ and NaCl in a mixture of CPME-H_2_O (Molina et al., 2012).

Using HCl as catalyst, water-MIBK biphasic system by mixing water phase with DMSO and butanol was reported (Chheda et al., 2007). The results showed that furfural yield from D-xylose increased (65 vs. 29%) with a 6-fold improvement in dehydration rate by decreasing pH (1.0 vs. 2.0) in the presence of water-DMSO mixture (5:5, wt/wt) and MIBK-2-butanol (7:3, wt/wt) as an extracting solvent. With xylan as feedstock, 66% of furfural could be achieved at pH = 1.0 in the same conditions. However, in water-DMSO mixture (3:7, wt/wt) and dichloromethane (DCM) as an extracting solvent at 140°C without catalyst, 57 and 76% of furfural yielded from D-xylose and xylan respectively in 3 h. Although the large-scale use of DCM would be restricted due to environmental concerns, this system showed promising conditions to solve the corrosion problem caused by adding mineral acids and effectively deal with insoluble and soluble biomass feedstocks. The evaluation of MIBK efficiency in enhancing furfural yields was also studied (Zhang T. et al., 2013). Using maple wood solid particles (5 wt%) in aqueous sulfuric acid (0.1 M) and water-MIBK mixture (1:1, wt/wt), furfural yield can reach 85% at 170°C for 50 min, which is better compared to the result achieving less than 65% of furfural yield in absence of MIBK extraction and just over 67% with hydrochloric acid catalysis for 60 min. Interestingly, when monosaccharide as D-xylose was chosen as feedstock, HCl revealed more efficient as acid catalyst with about 76% of furfural yield. This result remained superior to the yield of 64% with H_2_SO_4_ at 170°C for 30 min, simultaneously with MIBK as extraction solvent. In 2017, the production of furfural from 8 wt% pentose-rich corn stover hydrolyzate was reported (Mittal et al., 2017). The optimized procedure employed a diluted aqueous sulfuric acid solution (0.05 M) and the mixture was heated up at 170°C for 20 min under conventional heating in presence of MIBK. The D-xylose content was entirely converted into furfural with 80% yield. CPME as an efficient eco-friendly co-solvent was used for the selective dehydration of lignocellulosic pentose to furfural (Molina et al., 2012). They have clearly proven that CPME leads to nearly 100% furfural selectivity from lignocellulosic pentose under the following reaction conditions: H_2_SO_4_ (1 wt%), biomass (4 wt%) referred to aqueous solution at 170°C for 30 min, CPME/aqueous phase mass ratio equal to 2.33, and NaCl/aqueous solution mass ratio of 0.4 (Scheme 5). Like other organic co-solvents, CPME not only favors high furfural selectivity, but also prevents furfural degradation by keeping it in organic phase. In this study, NaCl addition was proved to play the role of accelerating furfural formation rate and shortening the reaction times.

In water-toluene biphasic system, the dehydration of D-xylose to furfural with H_2_SO_4_ as catalyst in association with an inorganic salt such as NaCl or FeCl_3_ as promoter was described (Rong et al., 2012). The maximum yield of furfural (83%) could be achieved in their work when NaCl (2.4 g) was added to water-toluene mixture (10:150, v/v) with D-xylose (10%) and H_2_SO_4_ (10%), while heating the mixture at the boiling temperature for 5 h. As a promoter, FeCl_3_ revealed better than NaCl. Aromatic solvent such as alkylphenol derivatives permitted the conversion of hemicellulose to furfural and levulinic acid using biphasic reactors with alkylphenol solvents that selectively partitioned furanic compounds from acidic aqueous solutions (Gürbüz et al., 2012). Then, 2-sec-butylphenol was identified as a new extracting solvent for the effective extraction of levulinic acid from acidic aqueous solution, and also for extracting furfural from aqueous phase with an exceptionally high partition coefficient when aqueous phase is saturated with NaCl. Some results of furfural production from D-xylose and corn stover from reported studies are summarized in Table 1.

**Table 1:** Conditions and results for the D-xylose dehydration with HCl as catalyst in an aqueous NaCl and 2-sec-butylphenol mixture (Gürbüz et al., 2012).

**Entry^a^**	**D-xylose (wt%)**	**HCl (M)**	***t* (min)**	**Conv. (%)**	**Sel. (%)**	**Yield (%)**
1	1.5	0.1	20	98	80	78
2	1.1 (from corn stover)	0.1	30	95	74	70
		0.25	15	92	82	75
3	5	0.1	15	92	77	71
4	2.1 (2 cycles from corn stover)	0.25	15	95	75	71

a *Reaction conditions: HCl (0.1–0.25 M), saturated aqueous NaCl-2-sec-butylphenol mixture (6.67:1, wt/wt), 170°C*.

Alginate could yield furan compounds after acidic treatment at elevated temperature. In this regard, alginic acid direct catalytic conversion to furfural using 12-tungstophosphoric acid (HPA) and H_2_SO_4_ as catalyst was investigated (Park et al., 2016). HPA exhibited higher catalytic activity than H_2_SO_4_, and gave the highest furfural yield (34%) at 180°C for 30 min. THF was found more suitable than water as reaction solvent, and the addition of a certain amount of water induced a synergistic effect, enhancing the production of furfural.

Microwave irradiation as an alternative technology was studied in the presence of HCl in the water-MIBK mixture together with the delivery of a kinetic model for the dehydration of D-xylose to furfural in a biphasic batch reactor (Weingarten et al., 2010). It was demonstrated that the organic phase, here MIBK, only acted as “storage” for the extracted furfural improving the furfural yield. However, the biphasic system does not alter the fundamental kinetics compared to the current monophasic system. Moreover, microwave heating does not change the kinetics compared to conventional heating technique. Under optimal reaction conditions: D-xylose (10 wt%) and aqueous HCl (0.1 M) at 170°C for ~70 min in a water-MIBK solution (1:1, wt/wt) as a biphasic system, furfural yield can reach 85%, which is more than two-fold of that obtained in monophasic system (30%) under the same conditions.

**Scheme 6:**
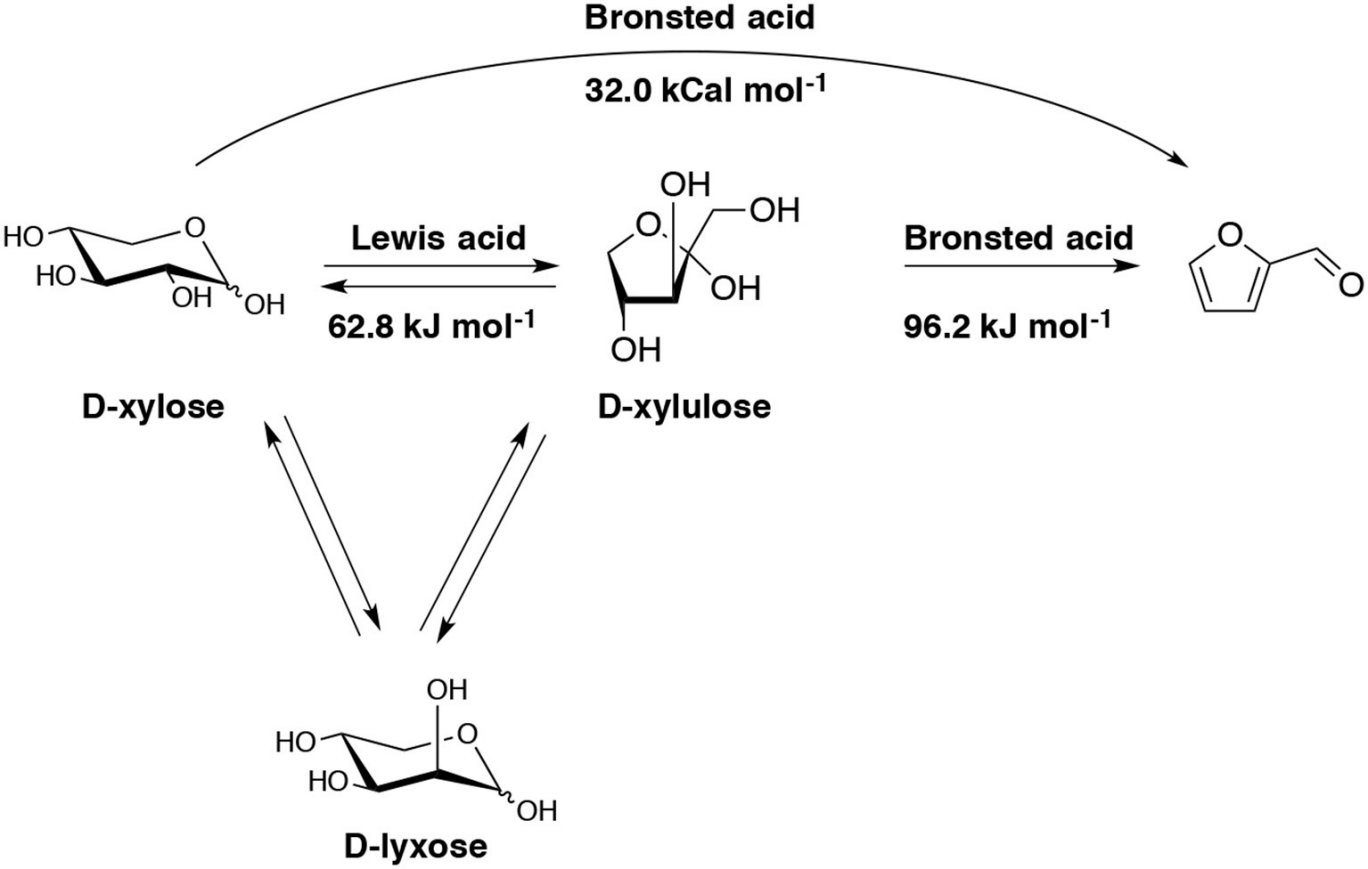
Schematic representation of the overall pathways to produce furfural from D-xylose in the presence of a single Brønsted acid catalyst or both Lewis and Brønsted acid catalysts (Choudhary et al., 2012).

The role of molecular structure on pentose dehydration to furfural has been examined using HCl as a Brønsted acid catalyst in a single phase aqueous media (Choudhary et al., 2012). It appears that the dehydration of D-xylose in the presence of Brønsted acid follows a direct path (Scheme 6).

When combined Lewis acid with Brønsted acid, D-xylose could isomerize to D-xylulose and D-lyxose by Lewis acid and subsequently dehydrates to furfural (Table 2). With this combined catalyst functionalities, a much higher yield (76%) to furfural can be obtained in a biphasic system at low temperatures and with short times.

**Table 2:** Process conditions and results for the dehydration of D-xylose with HCl and CrCl_3_, 6H_2_O and different solvents (Choudhary et al., 2012)^a^.

**Entry**	**Catalyst**	**Solvent**	**Time (min)**	**Conv. (%)**	**Yield (%)**
1	HCl (0.1 M)	Water	300	76	29
2	HCl (0.1 M) CrCl_3_, 6H_2_O (2 mM)	Water	150	75	32
3	HCl (0.1 M) CrCl_3_, 6 H_2_O (6 mM)	Water	90	90	38
4	HCl (0.1 M) CrCl_3_, 6H_2_O (13.5 mM)	Water	60	96	39
5	CrCl_3_, 6H_2_O(6mM) +HCl (0.1 M)	Water + Toluene	120	96	76
6	HCl (0.1 M)	Water + Toluene	120	31	27
7	CrCl_3_, 6H_2_O (6 mM)	Water + Toluene	120	96	35

a *Reaction conditions: Initial D-xylose 1 wt% (1 mL of aqueous solution for entry 1-4, 2 mL of aqueous solution and 2 mL toluene for 5-7), HCl (0.1 M) and/or CrCl_3_, 6H_2_O (2–13.5 mM), 145^o^C*.

An interesting study compared the furfural yields obtained respectively from the dehydration of D-xylulose and D-xylose in presence of H_2_SO_4_ under microwave heating in a range of temperature found between 180 and 220°C (Ershova et al., 2015). According their kinetic model, one conclusion is that the D-xylose isomerisation in these conditions is not a crucial step in the pentose dehydration process.

### Dehydration in presence of organic acids

Although mineral acids are widely used organic acids are suitable catalyst candidates toward minimization of corrosion issues. The mostly used organic acids for the production of furfural are formic acid and methanesulfonic acid. The comparison of the catalytic action of acids such as formic acid, sulfuric acid and phosphoric acid for the dehydration of D-xylose to furfural at temperature range from 135 to 200°C was reported (Yang W. et al., 2012). Formic acid performed the best furfural yield of 70% at 180°C against 62 and 65% for H_2_SO_4_ and H_3_PO_4_ respectively, both set at 160°C. Response surface methodology was applied to optimize furfural yield and selectivity with formic acid as the catalyst, and the best furfural yield reached 74% with D-xylose (40 g/L) and formic acid (10 g/L) initial concentrations, at 180°C. The effect of Kraft-lignin on acid-catalyzed D-xylose to furfural in formic and sulfuric acid using D-optimal design showed that lignin has an acid-neutralizing capacity and a negative effect on furfural formation from D-xylose (Lamminpää et al., 2015). It has been shown that at lower temperature, the yield of furfural is a bit better in the presence of lignin than without lignin in formic acid. Furthermore, the effects were greater in H_2_SO_4_ than in formic acid. The same group also investigated the kinetics of formic acid-catalyzed D-xylose dehydration into furfural and furfural decomposition, using batch experiments within a temperature range of 130–200°C (Lamminpää et al., 2012). By comparing three kinetic models, it is clear that the prediction model must consider other reactions besides furfural formation in overall D-xylose acid-catalyzed conversion. Moreover, the reactions between D-xylose intermediates and furfural play only a minor role. The study also showed that the pH of the reactant solutions has more effect on the reaction rate of furfural decomposition when temperature rises, thus, the kinetic modeling of the D-xylose and furfural decomposition reactions should be considered in water. AlCl_3_ as additive was used to improve the role of formic acid as catalyst. The double effect of a catalytic combination of AlCl_3_ with HCOOH on the furfural dehydration was studied (Lopes et al., 2017). Herein, the production of furfural was permitted by D-xylose isomerisation into more reactive D-lyxose in presence of the Lewis acid before the dehydration by the organic acid. The optimized condition involved an aqueous solution of AlCl_3_ (0.4 M) mixed with formic acid (55 wt%). The furfural selectivity reached a value of 74% when the reaction was carried out at 130°C.

Furfural was produced with a yield of 36% directly from oil palm fronds using formic acid as catalyst when immersed in ethanol under supercritical conditions (Yong et al., 2016). Higher reaction temperature taken between 240 and 280°C showed a great impact on the furfural yield. The supercritical ethanol had a significant role and the highest yield was obtained for an alcohol-acid ratio of 1:2, 0.4 g of solid loading for a mixture heated at 280°C for 20 min.

The use of another organic acid such as methanesulfonic acid was reported in presence of other additives. Under optimized D-xylose (0.3 M) and methanesulfonic acid (0.25 M) loading, furfural yields reached values of 60% at 160°C for 60 min, 65% at 180°C for 15 min and 63% at 200°C for 8 min, which are similar with the results of 66, 62, and 64%, (respectively for same temperature and duration conditions) with H_2_SO_4_ (Rackemann et al., 2014). The results indicated that methanesulfonic acid is a promising alternative catalyst to H_2_SO_4_ for the production of furfural from D-xylose. It should be noted that the addition of D-glucose into D-xylose had a significant impact on furfural yield, leading to lower yield values in that case with both designated catalysts. For example, furfural yield catalyzed with methanesulfonic acid decreased (45 vs. 63%) as mentioned above with D-glucose addition (0.1 M), and decreased more (41 vs. 64%) with H_2_SO_4_. It was considered that D-glucose addition resulted in the promotion of furfural degradation. The same group produced furfural in high yield from exposure of pretreated sugarcane bagasse with methanesulfonic acid but the target furanic compound was obtained in association with larger amount of levulinic acid (Rackermann et al., 2016). The use of ionic liquids (IL) in presence of methanesulfonic acid was reported starting from lignocellulosic biomass (Vanoye et al., 2009). In this work, 25% of furfural yield could be obtained at 100°C for 30 min starting from biomass-miscanthus which consist of 44% cellulose and 24% hemicellulose (0.1 g/cm^3^) in the presence of 1-ethyl-3-methyl-imidazolium chloride (5 mL), methanesulfonic acid (0.11 mL), water (3 mmol/cm^3^). It should be noted that recent works described thermal and combustion risk profiles of ILs as well as risks of toxicity (Diallo et al., 2012a,b, 2013; Chancelier et al., 2014; Bado-Nilles et al., 2015) and consequently a study of the stability and fate of the ILs in the process should be studied.

In the optic to have a greener process, different biobased compounds obtained in the biorefinery have been tested as acid catalyst. Biobased maleic acid has been used as an efficient catalyst to convert xylan to D-xylose at high yields in aqueous solution at relatively mild temperature (160°C), and to subsequently dehydrate the resulting D-xylose to furfural (Kim et al., 2012). The kinetics of D-xylose dehydration to furfural using maleic acid predicted maximum furfural yield of 72%, while the observed yield was 67% at 200°C for 28 min with microwave heating. In 15 min, furfural yields ranging in 54 to 61% could be achieved with poplar, switchgrass and corn stover respectively as feedstocks, in comparison to a furfural yield not exceeding 29% from pine. Maleic acid also showed some promising advantages in regard to reusability, although maleic acid is slowly hydrated to malic acid.

**Scheme 7:**
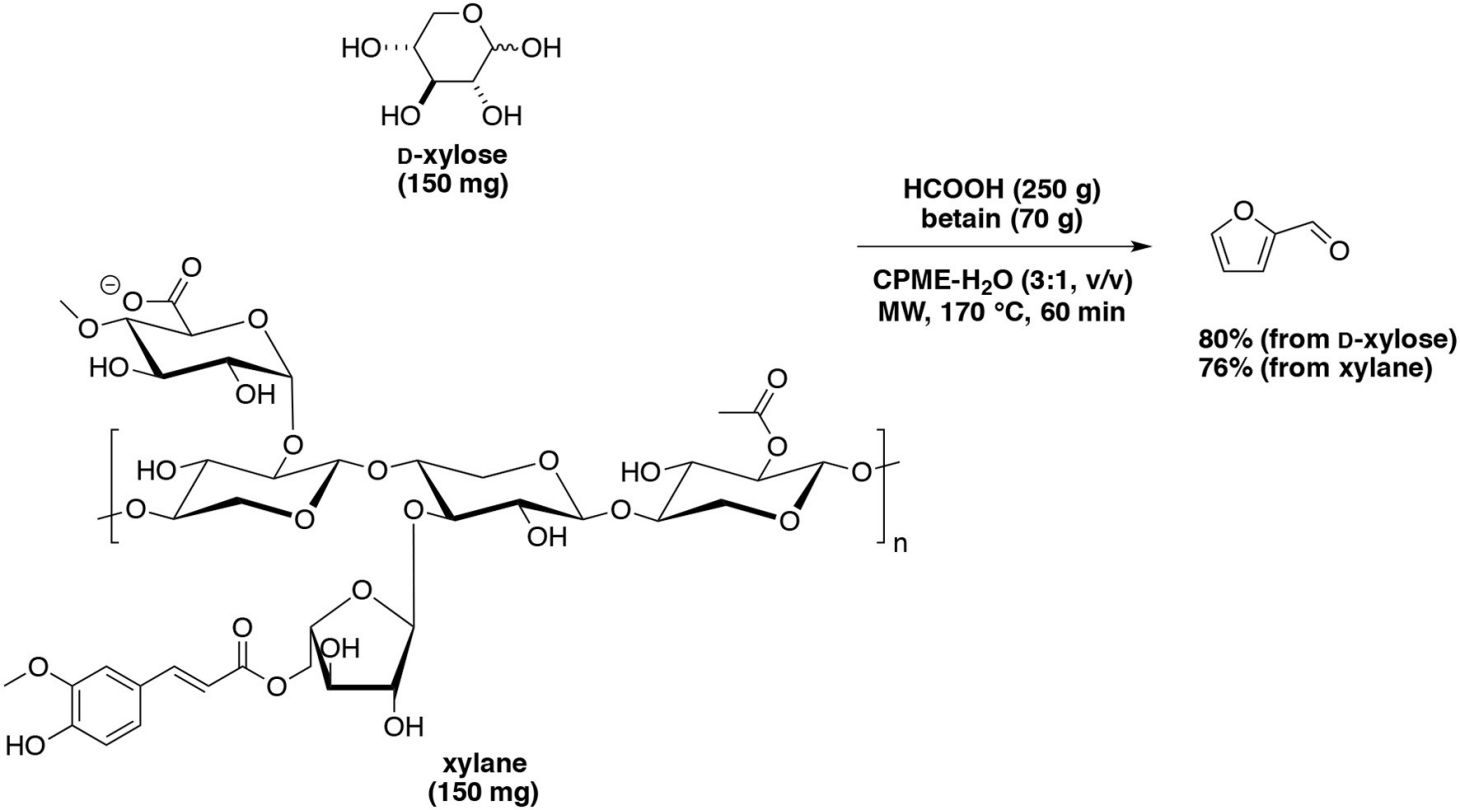
Microwave-assisted production of furfural from D-xylose in the presence of formic acid and betain in a mixture of CPME-H_2_O (Delbecq et al., 2016).

As mentioned above, the addition of organic solvents helps the extraction of furfural. Using organic catalysts, the following process was also studied. o-Nitrotoluene acted as extraction solvent for furfural production (Yang et al., 2013). The maximum furfural yield of 74% and selectivity of 86% were obtained in 75 min when heated at 190°C starting from D-xylose (80 g/L) and formic acid concentration (20 g/L) with 75% v/v o-nitrotoluene. Additionally, the use of salts (KI, KBr, KCl, and NaCl) was able to enhance the furfural yield and selectivity, whatever their concentration. Len's group demonstrated that formic acid combined with betaine chloride was an efficient and novel homogeneous catalytic system for direct transformation of D-xylose and xylan into furfural (Delbecq et al., 2016). Under optimized conditions at 170°C for 1 h, 80% and 76% of furfural yields were respectively achieved from D-xylose and xylan in a CPME-water biphasic system under microwave-assisted heating (Scheme 7). Other mixtures of solvents such as water-GVL and water-2-methyltetrahydrofurane (MTHF) were used for the dehydration of D-xylose (Xu Z. et al., 2015). Furfural yield (59%) was achieved in the presence of *p*-toluenesulfonic acid for 10 min at 170°C with D-xylose (0.4 g) in a mixture of water-GVL (1.5:15, v/v).

Levulinic acid was explored as a biobased catalyst to produce furfural from pinewood and eucalyptus sawdust in a water or water-MTHF biphasic system (Seemala et al., 2016). Using levulinic acid as the catalyst benefited to both hydrolysis and dehydration processes due to its solubility in the water and MTHF phase. As expected, biphasic system was superior to mono-system. In the presence of feedstock (0.4 g), levulinic acid (0.1 g) and water-MTHF (1:1, wt/wt) loading, eucalyptus isomer revealed to be an easier biomass to convert into furfural than pinewood (565 mg/L at 180°C for 15 min vs. 643 mg/L at 200°C for 60 min), in spite of the fact that eucalyptus gave less C5 sugars which could be due to the different composition of hemicelluloses.

### Dehydration in presence of metal salts

Different metal salts have been studied for the production of furfural in a monophasic and biphasic system. Dehydration of xylan and D-xylose to furfural with Cr (II) or Cr (III) as catalysts and *N,N*-dimethylacetamide (DMA) as solvent was reported (Binder et al., 2010). At 100°C, D-xylose was converted into furfural in 30–40% yield in DMA using CrCl_2_ and CrCl_3_. However, when LiBr was added as an additive, furfural yield catalyzed with CrCl_2_ increased up to 56% and 47% if CrCl_2_ is substituted by CrCl_3_. Other work on a copper catalyst showed that Cu (II) is the most efficient among various metal cations for alginic acid hydrothermal treatment to produce furfural. In this work, a yield of furfural of 13% was obtained at 200°C for 30 min (Jeon et al., 2016a). In spite of low catalytic performance for degrading alginic acid, Cu (II) ions are favorable for the conversion of alginic acid to furfural. This study implied that algae derivative such as alginic acid could be potentially used as a sustainable alternative feedstock for the production of furfural in the future.

Sodium molybdate as metal salt in presence of HCl as acid co-catalyst permitted the stereospecific conversion of D-xylose and D-lyxose from xylan especially while combined with microwave technology (Hricovíniová, 2013). Simultaneously the homogeneous catalyst leads to higher energy transfer to solution from a microwave source that will improve the yield of furfural. The process involves combined hydrolysis, epimerization and dehydration reactions in a single step and provided higher amounts of furfural (53%) compared to reaction without Mo (VI) ions (42%, 300 W for 5 min of MW heating). Xylan dehydration with conventional heating in the presence of molybdate yielded 36% of furfural that is also higher than the 28% yield obtained without Mo (VI) ions at 150°C for 30 min. It was proposed that chromium, acting as a Lewis acid, catalyzed D-xylose isomerization (Choudhary et al., 2012). In fact, CrCl_3_ is an efficient Lewis acid to isomerize D-xylose to D-xylulose and further dehydrated it to furfural (76%) at lower temperature (145°C). When xylan was used as the feedstock, the results implied that depolymerization is the major barrier for chromium-catalyzed furfural production, and less than 25% of furfural was achieved at 140°C with CrCl_2_ as catalyst, HCl as co-catalyst in 1-ethyl-3-methylimidazolium chloride.

Focusing only on the use of NaCl as catalyst, a comparison was also made between conventional heating and microwave technology (Xiouras et al., 2016). Higher yields were obtained after 7 min of heating in presence of NaCl (3.5 wt%) at 200°C. The D-xylose conversion was complete giving 76% of furfural yield under microwave heating.

Using metal salts, biphasic systems were developed making use of similar solvents as mentioned above. Isolation of hemicelluloses from Rubescens using a water-GVL biphasic system heated at 180°C was studied (Luo et al., 2017). The hemicelluloses rich lignin fraction was then warmed up in presence of NaCl and THF giving a furfural yield of 77%. NaCl was also found responsible of all reactions such as hydrolysis, epimerization and dehydration involved in the processes at the higher temperature of 200°C. A production of furfural was set up in association with bromomethylfurfural (BMF) and HMF from cellulose and lignocellulosic biomass (Yoo et al., 2017). The biphasic system water-DCM (1:3, v/v) involving an organic solvent and a molten lithium bromide hydrate solution was heated for 2 h at 120°C and permitted to obtain furfural in 70% yield. The use of high pressure of CO_2_ in a mixture of aqueous isopropanol in the presence of NaCl furnished furfural in 70% yield (Zhao et al., 2017). The high CO_2_ concentration reduced the pH of the sugar solution and the optimized condition required a temperature of 200°C for 3 h in a 2:1 ratio of water-alcohol. Different works described the use of iron-catalyzed furfural production in biobased biphasic systems. FeCl_3_ was selected as the catalyst, NaCl as additive and MTHF as biomass-derived solvent (Vom Stein et al., 2011). Without NaCl, furfural yield only reached 27% with FeCl_3_ as catalyst and a reaction temperature of 140°C, however, the same reaction condition involving NaCl (20 wt%) for 4 h afforded furfural (70%). Furfural formation rate increased by a factor of more than two. Xylan from beech wood was first hydrolyzed to D-xylose in a concentration of 30 g/L (2.5 mL), then FeCl_3_, 6H_2_O (0.12 M), NaCl (30 wt%), were added together in 2.5 mL of MTHF, and the reaction yielded furfural (37%) at 140°C for 2 h. Furfural yield of 75% was obtained from D-xylose in a water-THF biphasic medium containing AlCl_3_, 6H_2_O and NaCl under microwave heating at 140°C (Yang Y. et al., 2012). It was clear that AlCl_3_ could isomerize D-xylose into D-xylulose, followed by the dehydration of the latter to produce furfural, similarly to isomerization catalysts CrCl_3_ or NaMoO_4_ mentioned above. Moreover, this system was effective in the hydrolysis of xylan and lignocellulosic hemicelluloses to D-xylose, which resulted in fairly good furfural yields in some cases from lignocellulosic biomass (Table 3).

**Table 3:** Conditions and results for the production of furfural starting from various sources of lignocellulosic biomass in presence of AlCl_3_ (Yang Y. et al., 2012).

**Entry^a^**	**Biomass**	**Temperature (°C)**	**Time (min)**	**Yield (%)**	
				**D-Furfural**	**D-Xylose**
1	Corn stover	140	60	51	2
2	Pinewood			29	12
3	Switchgrass			50	8
4	Poplar			45	6
5	Corn stover	160		55	<1
6	Pinewood			38	<1
7	Switchgrass			56	1
8	Poplar			64	<1
9	Pinewood	180	30	61	<1

a *Reaction conditions: Biomass 0.05 g, AlCl_3_, 6H_2_O 0.1 mmol, NaCl 6.0 mmol, water 1 mL, THF 3 mL*.

**Scheme 8:**
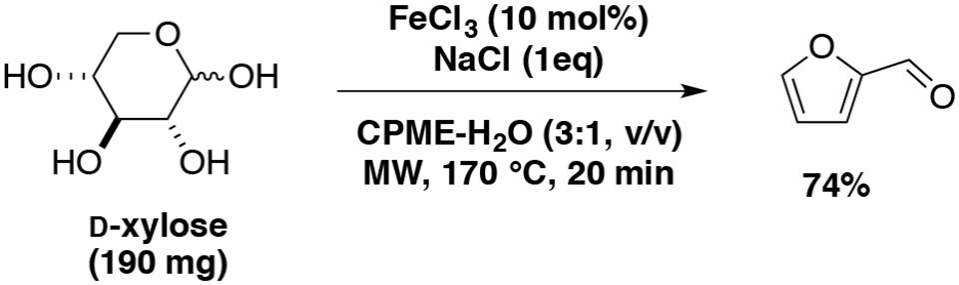
Microwave-assisted production of furfural from D-xylose in the presence of FeCl_3_ and NaCl in a mixture of CPME-H_2_O (Le Guenic et al., 2015).

Starting from D-xylose, our team also studied furfural production catalyzed with FeCl_3_ and NaCl as additive (Le Guenic et al., 2015). With the aid of microwave irradiation and the eco-friendly mixture: water-CPME (1:3, v/v), the highest furfural yield (74%) was achieved at 170°C for 20 min in the presence of iron chloride (10 mol%) and NaCl 100 (mol%) (Scheme 8). Addition of NaCl was found to increase the catalytic activity of FeCl_3_ and allowed to reduce the amounts of FeCl_3_ used from 20 mol% to 10 mol%. This system was extended to xylan and afforded furfural yield of 52% at 200°C for 20 min or 170°C for 70 min.

Sulfate derivatives such as NaHSO_4_, KAl(SO_4_)_2_ and Al_2_(SO_4_)_3_ were tested for the production of furfural. Furfural was obtained from various raw lignocellulosic materials in water-THF by using NaHSO_4_ as catalyst (Shi et al., 2015). Many reaction parameters were optimized, such as reaction temperature, time, solvent volume ration, feedstock concentration as well as catalyst loading. Under the optimum conditions: 190°C, 90 min, biomass concentration (11.1 wt%), NaHSO_4_ (3.31 wt%), H_2_O (0.8 mL) and THF (8 mL), 50-60% furfural yields were obtained from diversified lignocellulosic biomass such as corncob, wheat straw, bagasse. Under conventional heating, using KAl(SO_4_)_2_ in a water-MIBK biphasic system, furfural was efficiently produced from D-xylose when heated for 6 h at 190°C, since 55% of furfural yield was obtained (Gupta et al., 2017a). The catalytic activity of Al_2_(SO_4_)_3_ metal salt for the D-xylose dehydration was also reported using a water-GVL biphasic system (Yang et al., 2017). Al_2_(SO_4_)_3_ decomposes into ${\text{SO}}_{4}^{2-}$ anions and the hexacoordinated Lewis acidic species [Al(OH)_2_(HO)_4_] able to isomerizes D-xylose into D-xylulose, easily converted into furfural. The optimized conditions afforded 88% yield in furfural

**Scheme 9:**
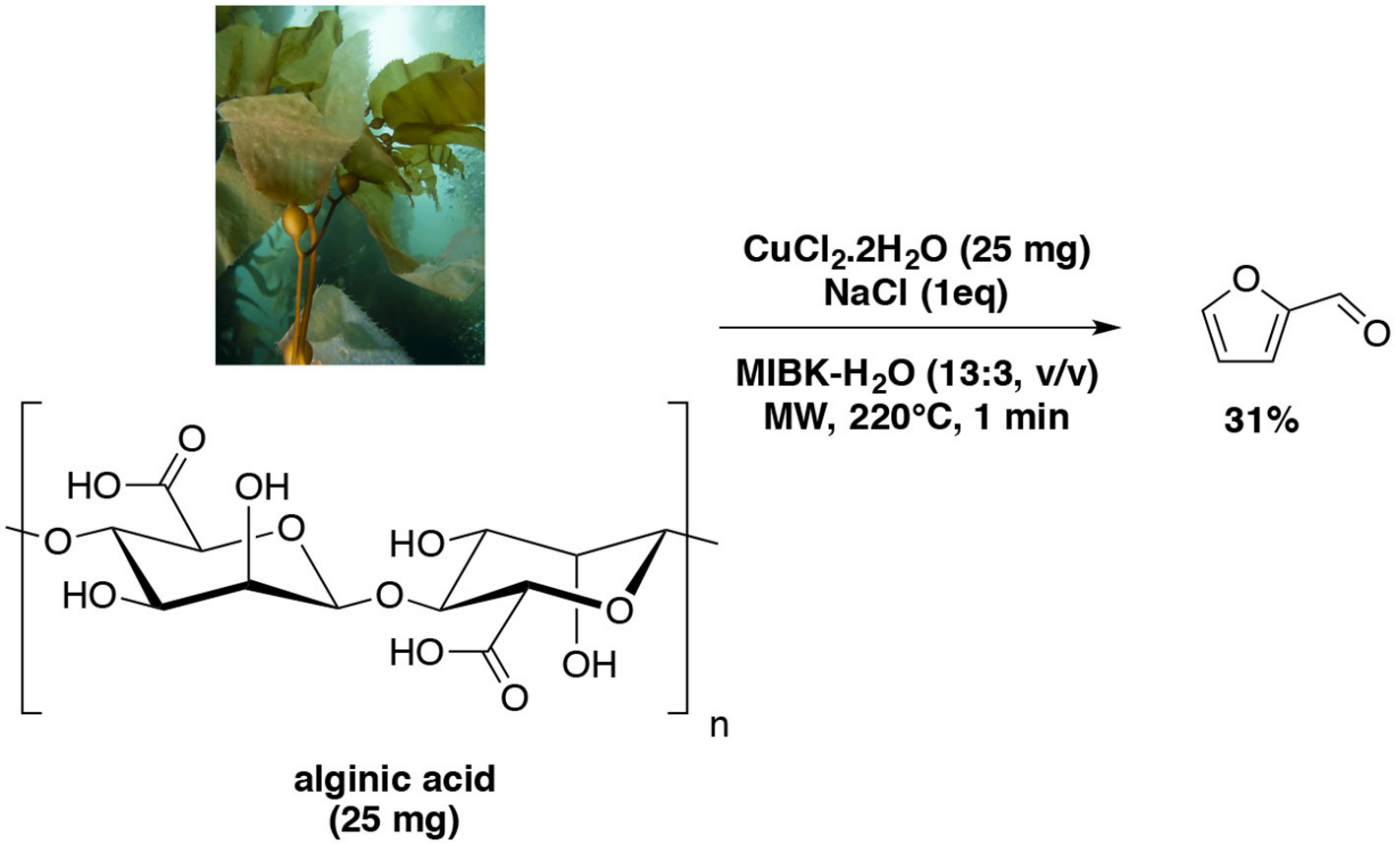
Microwave-assisted production of furfural from D-xylose in the presence of CuCl_2_.2H_2_O in a mixture of H_2_O-MIBK (Wang et al., 2018).

Interestingly, a most recent work reported an improvement of the furfural yield from alginic acid also catalyzed by Cu (II) as previously reported in various biphasic system (Wang et al., 2018). With MIBK, the microwave-aided dehydration of the polysaccharide afforded furfural in an overall yield of 31% (hydrolysis, decarboxylation, dehydration) (Scheme 9).

### Ionic liquids (ILs) applications in catalysis for furfural synthesis

ILs are known as organic salts with melting points below 100°C composed solely of cations and anions, which have many unique properties, such as very low volatility, good dissolving capacity, operational chemical and thermal stability, flame-retardancy (often leading to qualifying them as “non-flammable” products as resulting from the application of haz-mat regulations. Some are non-toxic, but not all of them and recyclability is more or less easy. ILs are often considered -sometimes too generically- as an example of more sustainable solvents, which contribute to a greener processing of biomass, and are suitable for furfural production (Lima et al., 2011). Some neutral ILs such as 1-butyl-3-methylimidazolium chloride (Sievers et al., 2009; Zhang L. et al., 2013; Peleteiro et al., 2014) has been employed as efficient solvents and 1-ethyl-3-methylimidazolium chloride used as additive (Binder et al., 2010). Generally, acidic ILs were applied as catalysts which performed as reaction mediums (Peleteiro et al., 2016a), such as 1-butyl-3-methylimidazolium hydrogen sulfate (Carvalho et al., 2015; Peleteiro et al., 2015a,b), 1-ethyl-3-methylimidazoliumhydrogen sulfate (Lima et al., 2009), 1-(4-sulfonic acid) butyl-3-methylimidazolium hydrogen sulfate (Tao et al., 2011, 2012). Even though ILs have showed many advantages for the production of furfural, scientists are also facing plenty of challenges to realize their industrial utilization, such as the cost of ILs, deep understanding of their properties, their recovery and recycling problems and also environmental issues. Meanwhile, the recovery of furfural from IL should also become difficult. Moreover, our group reported that ILs could generate fire induced toxicity essentially driven by the presence of hetero-atoms in their structures and induce ecological disturbance for organism (Diallo et al., 2012a,b, 2013; Chancelier et al., 2014; Bado-Nilles et al., 2015). Thermal stability advantages of ILs need also to be analyzed on a case by case study (Abbott et al., 2018). In order to limit the use of ILs, different groups reported the production of furfural with acid ILs used as reagent and not as solvent (Matsagar et al., 2015; Peleteiro et al., 2016b,c; Liu et al., 2017; Matsagar and Dhepe, 2017; Wang et al., 2017).

## Synthesis of furfural from sugars and polysaccharides using solid acid catalysts

Production of furfural starting from pentose derivatives has been reported using carbon acids, clays, ion-exchange resins, oxides, phosphates, silicates and zeolithes sometimes assisted by alternative technologies (Bhaumik and Dhepe, 2016).

### Dehydration in presence of carbon acids

Carbonaceous materials are frequently used solid acid catalysts in many applications due to their high thermal stability, high chemical activity and low production costs. Therefore, several researchers have considered that they could be an interesting alternative for sugar dehydration in aqueous environment. An activated carbon catalyzed D-xylose dehydration to furfural was compared with an auto-catalytic reactions (Sairanen et al., 2014). The use of Norit as commercially-available activated carbon permitted to have a better control over the unwanted side reactions such as acids and humins formations. It was found that the main isomer with carbon catalyst was D-xylulose and in case of non-catalyzed reaction was D-lyxose. This result demonstrated that activated carbon catalyst could avoid unwanted side reactions by offering acidic sites and could permit isomerization of D-xylose into more reactive keto sugars, these latter intermediates being more favorable to furfural production. Other carbon sources such as graphene derivatives were studied for the production of furfural to improve the conversion and selectivity. In this regard, four kinds of carbon-based catalysts: graphene, graphene oxide, sulfonated graphene, and sulfonated graphene oxide (SGO) were elaborated and tested (Lam et al., 2012) (Table 4).

**Table 4:** Conditions and results for the production of furfural starting from D-xylose and grapheme derivatives in water (Lam et al., 2012).

**Entry^a^**	**Catalyst**	**Conv (%)^b^**	**Sel. (%)^b^**	**Yield (%)^b^**
1	None	76	58	44
2	Graphene	75	68	51
3	Graphene oxide	80	66	53
4	Sulfonated graphene oxide	83	75	62
5	Sulfonated graphene	86	64	55

a *Reaction conditions: D-xylose (2.25 g), catalyst loading 2 wt%, 35 min, 200^o^C*.

b *Data averaged over 3 runs*.

SGO was proven to be a rapid and an active catalyst for improving furfural yield from D-xylose aqueous solution even at very low catalyst loadings down to 0.5 wt% vs. D-xylose. Furthermore, SO_3_H groups, which are the active acidic sites for dehydration of D-xylose to furfural, have shown good water tolerance and revealed more thermally stable under the reaction conditions than COOH or OH groups. After 12 times repeated tests at 200°C for 30 min, an average furfural yield of 61% was achieved in comparison to 44% for the uncatalyzed system.

**Scheme 10:**
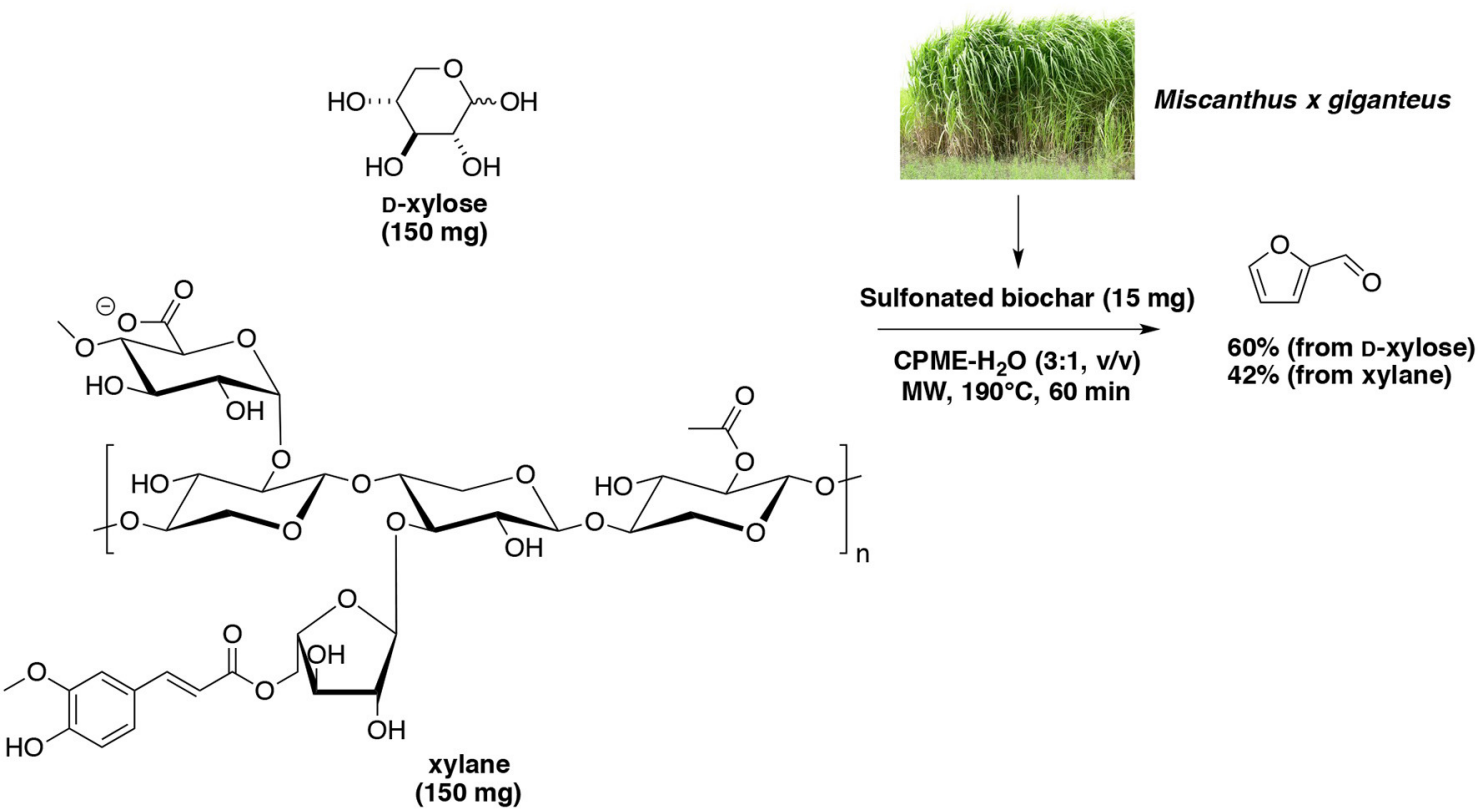
Microwave-assisted production of furfural from D-xylose in the presence of sulfonated biochar (ex Miscanthus x giganteus) in a mixture of H_2_O-CPME (Wang et al., 2017a).

Often the carbon-based catalysts are prepared by carbonization of sugar molecules to form sulfonate-functionalized carbon particles. In particular, solvothermal conversion of cassava waste to furfural using a sulfonated carbon-based catalyst was investigated (Daengprasert et al., 2011). Results obtained in presence of H_2_SO_4_ and without catalyst was compared, the carbon-based catalyst showed its effectiveness for the hydrolysis of xylan to D-xylose and then D-xylose dehydration to furfural. However, in this study, less than 3% of furfural yielded from cassava waste and only 12% from D-xylose and xylan. Sulfonated biochar was prepared successively by carbonization and sulfonation (Deng et al., 2016). The resulted acidic carbonaceous solid was employed for the direct transformation of a pre-hydrolyzed aqueous solution of corncob in a biphasic system involving DCM on the organic layer. When the system was heated at 170°C for 60 min, 83% of furfural selectivity was finally recorded. Len's group employed a sulfonated biochar in a microwave-aided dehydration of D-xylose and commercial xylan (Wang et al., 2017a). Each substrate was introduced in a water-CPME (1:3, v/v) biphasic system, and warmed up at 190°C for 60 min. Furfural was obtained with 60 and 42% of yield from D-xylose and xylan, respectively (Scheme 10).

Another source of carbon catalytic material was prepared (Zhang et al., 2016) by successively carbonization of sucrose and sulfonization, successively. This solid catalyst was able to dehydrate D-xylose and corn stalk into furfural at 170°C for D-xylose and 200°C for the plant waste. The yields obtained were 79 and 61%, respectively, for residence time varying between 30 and 100 min. Recently, a carbonized resorcinol-formaldehyde resin and sulfonated was also tried as a catalyst (Zhu et al., 2017). The material was found efficient for furfural production from D-xylose and corn stover in GVL. When heated at 170°C for 15 min with enough catalyst, the best dehydration conditions afforded furfural in 80% yield with a D-xylose conversion of 100%. In comparison, 69% of furfural yield was achieved from corn stover at 200°C after 100 min of heating with higher amount of the same catalyst. The sulfonation of carbonaceous catalyst SC-CCA prepared by carbonization of sucrose using 4-benzene-diazoniumsulfonate as a sulfonating agent produced furfural in 79% yield by use of GVL as unique solvent in a conversion reaction operated at 170°C for 30 min (Zhang et al., 2016). Starting from corn stalk, furfural was obtained in 60% yield for 100 min at 200°C or for 60 min at 210°C. In case of corn stalk, it is noteworthy that addition of water (10 wt%) severely decreased furfural yield. Nevertheless, sulfonated groups grafted onto the C-CCA have been demonstrated to be significantly stable, and SC-CCA could be reused up to 5 runs without the loss of furfural yields. Calcium citrate was used as novel source of biomass for the preparation of sulfonated catalyst (Li et al., 2017). The use of sulfonated carbonaceous residue obtained from the calcination of bio-based calcium citrate in dehydration reaction of raw corn stover in GVL operated at 200°C furnished furfural (93%) in GVL. The authors mentioned that using the same reactions conditions replacing GVL by water was leading to a furfural yield reaching only 51%.

**Scheme 11:**
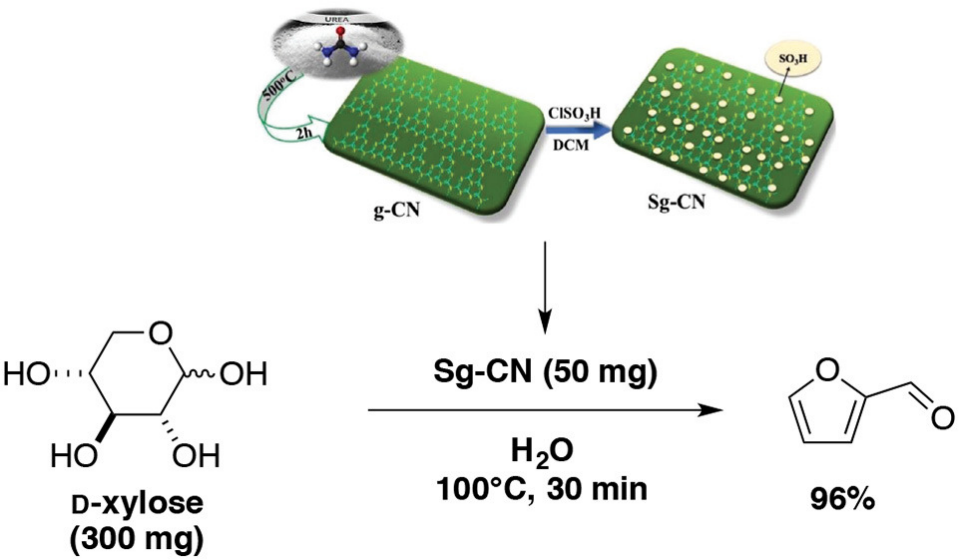
Production of furfural from D-xylose in the presence of sulfonated graphitic carbon nitride in water (Verma et al., 2017).

The preparation of a sulfonated graphitic carbon nitride and its application for the dehydration of D-xylose into furfural were reported by Varma and Len's groups (Verma et al., 2017). Starting from D-xylose in presence of sulfonated graphitic carbon nitride in water as sole solvent at 100°C for 30 min, the yield in furfural rose to 96% (Scheme 11). This process gave one of the best results obtained at the lab scale.

### Dehydration in presence of clays

Only one report described the use of clays for the dehydration of pentose derivatives into furfural. Four kinds of pillared clays catalysts with different quantities of aluminum and hafnium (labeled as Al-Hf 11-1, Al-Hf 10.5-1.5, Al-Hf 10-2 and Al-Hf8-4) were used and applied into the conversion of D-xylose to furfural (Cortés et al., 2013). The selectivity of furfural in this work reached 53% with Al-Hf 11-1 and increased to 65% with Al-Hf 10.5–1.5 in a reaction operated at 140°C; however, D-xylose conversion were only 45 and 35%, respectively by the way significantly limiting (below 30%) the overall yields in furfural obtained. Increasing reaction temperature to 170°C raised D-xylose conversion, but a loss of selectivity was observed. As a conclusion, an increase of the hafnium content reduced the selectivity for furfural on Al-Hf vermiculites. A maximum Hf content of 2 mmol Hf/g (clay) revealed however to be a promising catalyst for the dehydration of pentose in water as a solvent, producing furfural with an average conversion rate of 78% and a selectivity rate of 50% at 170°C for 4 h in four consecutive reactions.

### Dehydration in presence of ions-exchange resins and ionomers

Furfural production catalyzed by an ion-exchange sulfonic resin was extensively studied. The nature and properties of the resins *Amberlyst* and *Nafion*, with different acidities, pore diameters and thermal stabilities, permitted to generate the dehydrated chemicals depending of the process used. The kinetic parameters of furfural production in water and water-toluene biphasic system, with or without catalyst, at different reaction temperatures and time, D-xylose loading and its simultaneous stripping using nitrogen were studied (Agirrezabal-Telleria et al., 2011). In this work, 65% of the stripping furfural was achieved from D-xylose and almost 100% of selectivity in the condensate was observed. Later, the effect of D-glucose addition into D-xylose on furfural production was conducted under different operating configurations (Agirrezabal-Telleria et al., 2012). D-Glucose addition to D-xylose has a negative effect on furfural yield at 175°C, but a positive effect at higher temperature (200°C), which is considered as an “entropy-effect” at high reaction temperature, leading to slower side-reactions. The mixture of D-xylose and D-glucose at similar ratios to the real pentosan-rich biomass led to furfural yields of up to 75% at 200°C, and even 78% with lower D-xylose concentration. The same group showed that *Amberlyst-70* with strong sulfonic acid sites presented a higher furfural selectivity than Nb_2_O_5_ supported catalyst (Agirrezabal-Telleria et al., 2013a). In all their studies, the strategy of nitrogen stripping showed more industrial feasibility with respect to other biphasic water-solvent systems. Using the same catalyst, *Amberlyst-70*, a research aiming at producing specific humins has been done starting from D-xylose in a mixture of methanol-water (Hu et al., 2012). It was found that high reaction temperature, long residence time, low methanol-water mass ratio, and high catalyst dosage were favorable for their formation. Although furfural can be protected to form 2-(dimethoxymethyl)-furan (DOF) in the methanol-rich medium, this did not remarkably suppress polymerization occurrence at high reaction temperature. Additionally, the acid treatment of furfural also produced methyl levulinate in methanol and levulinic acid in water by partial degradation of furfural in those conditions. Later, further study of acid-treatment of C5 and C6 sugar monomers/oligomers with the same catalyst in water or DMSO system was conducted (Hu et al., 2014). Some interaction or cross-polymerization of D-xylose-D-glucose, D-fructose-raffinose, furfural-D-glucose, and furfural-D-fructose occurs. In water, yields of the insoluble polymer from the sugars / (furfural) increase in the order: D-fructose ~ raffinose > D-glucose > D-xylose > (furfural). In another hand, furfural can be preserved by DMSO and in such a case less insoluble polymer is formed.

The use of *Amberlyst-15* instead of *Amberlyst-70* has been applied to promote the catalytic hydrothermal reaction of alginic acid toward furfural production (Jeon et al., 2016b). A maximum of 19% furfural yield was obtained at 180°C in 30 min. The catalyst could be recycled 5 times with approximately 30% loss of furfural yield at latest catalyst recycling run. Unfortunately, recovered catalyst could not be regenerated with H_2_SO_4_ because by-products covering its surface inhibited its regeneration. In water-THF mixture, *Amberlyst-15* led to furfural production in 17% yield at reaction temperature of 170°C maintained for 60 min. The thermal stability of *Amberlyst-15* could explain this result. In comparison with the use of 12-tungstophosphoric acid poorer catalytic efficiency has been observed (Park et al., 2016). Successive dehydration of D-xylose by *Amberlyst-15* and furfural hydrogenation over a hydrophobic Ru/C catalyst in a single biphasic reactor have been studied (Ordomsky et al., 2013). Organic solvents butan-1-ol, MTHF and cyclohexane were used. The amounts of the catalysts, solvent, temperature and pressure were optimized in the water-cyclohexane system. The main product tetrahydrofurfuryl alcohol was obtained at 135°C with a selectivity and a conversion ratio of 50 and 32%, respectively. In addition, co-products such as GVL, levulinic acid and pentane diols were also observed with comparable yields.

**Scheme 12:**
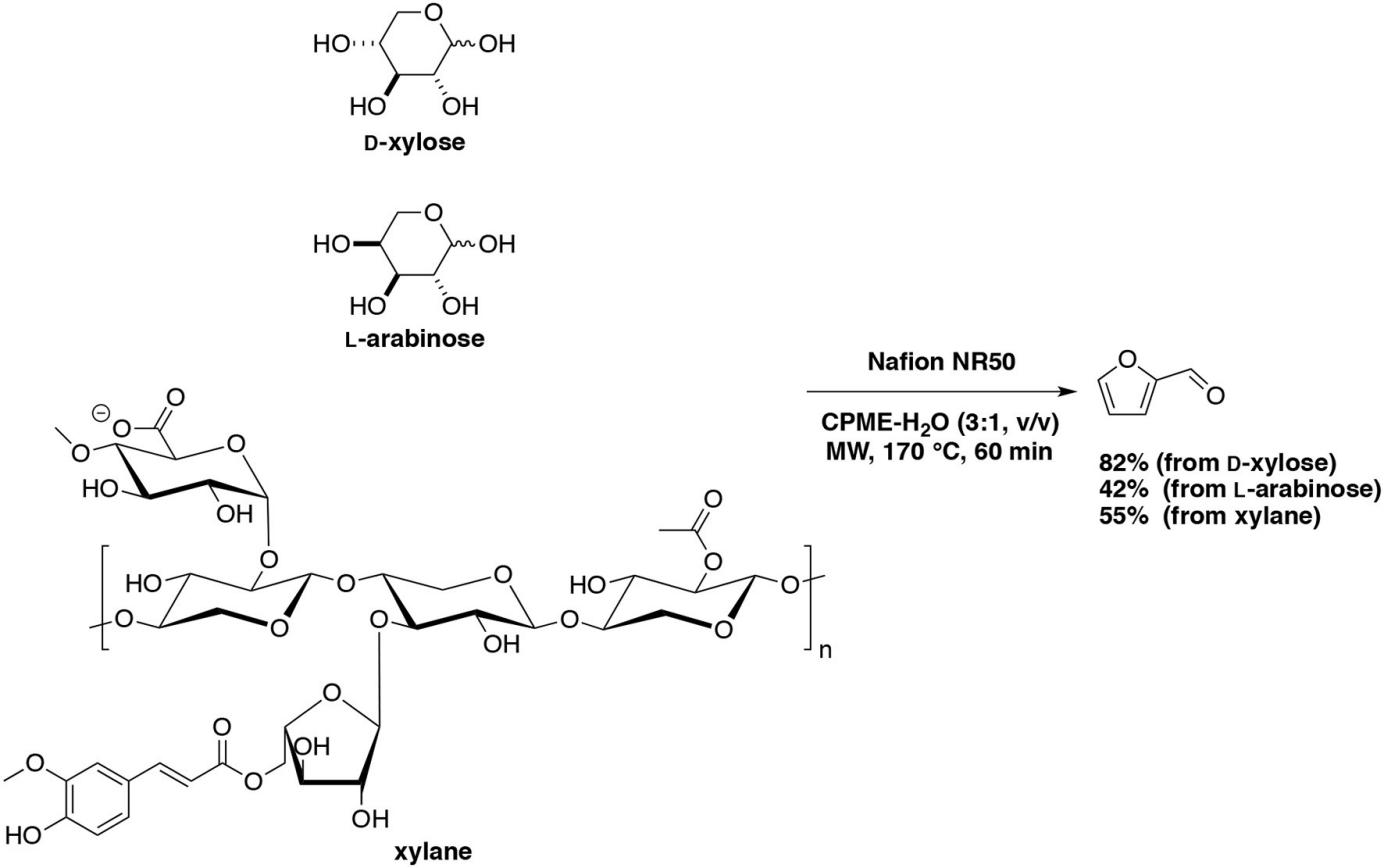
Microwave-assisted production of furfural from D-xylose, L-arabinose and lignin in the presence of *Nafion NR50* in a mixture of H_2_O-CPME (Le Guenic et al., 2016).

A more promising heterogeneous catalyst, *Nafion 117* furnished furfural in 58–62% yields in DMSO at 150°C and as a result of 15 consecutive runs with an overall conversion higher than 90% (Lam et al., 2011). Activation energy values for *Nafion 117* at 5 and 20% catalyst weight loading were 86.4 and 89.3 kJ/mol, respectively which were lower than other solid acid catalysts tested. Our group has tested perfluoroalkane sulfonic resin, *Nafion NR50*, anticipating a good efficiency as a superacid catalyst for the production of furfural (Le Guenic et al., 2016). In a water-CPME biphasic system under microwave irradiation D-xylose, L-arabinose and xylan converted into furfural in the presence of NaCl with maximum yields of 80, 42, and 55%, respectively (Scheme 12). It was found that the association of *Nafion NR50* and NaCl generated an unusual stability of the resin, which have synergistic effect in acid catalysis. Unfortunately, gradual deactivation due to humins deposition was also observed after the fourth cycle.

Three kinds of micro-mesoporous sulfonic acid catalysts-silylated MCM-41-SO_3_H, coated MCM-41-SO_3_H and hybrid-SO_3_H have been prepared, characterized, and tested in the dehydration of D-xylose to furfural (Dias et al., 2005b). The hybrid-SO_3_H gave a lower furfural selectivity compared to MCM-41-SO_3_H materials, which may be due to the hydrophobicity of the surface. This in turn might have enhanced the adsorption of furfural at the surface, and further accelerated its reaction with intermediates on the accessible acid sites. Coated MCM-41-SO_3_H yielded slightly higher yield (>75%) than MCM-41-SO_3_H (69%) in DMSO or water-toluene biphasic system at 140°C for 24 h. However, silylated MCM-41-SO_3_H have a better selectivity than that of coated MCM-41-SO_3_H (96 vs. 83%) in water-toluene. The increase of reaction temperature shortened the reaction time, and subquently benefited to the obtained furfural yield. For example, 70% of furfural yield was achieved at 170°C for 4 h with coated MCM-41-SO_3_H. Unfortunately, apparently catalyst deactivation was observed as soon as and the second catalyst reuse in spite of its regeneration.

**Scheme 13:**
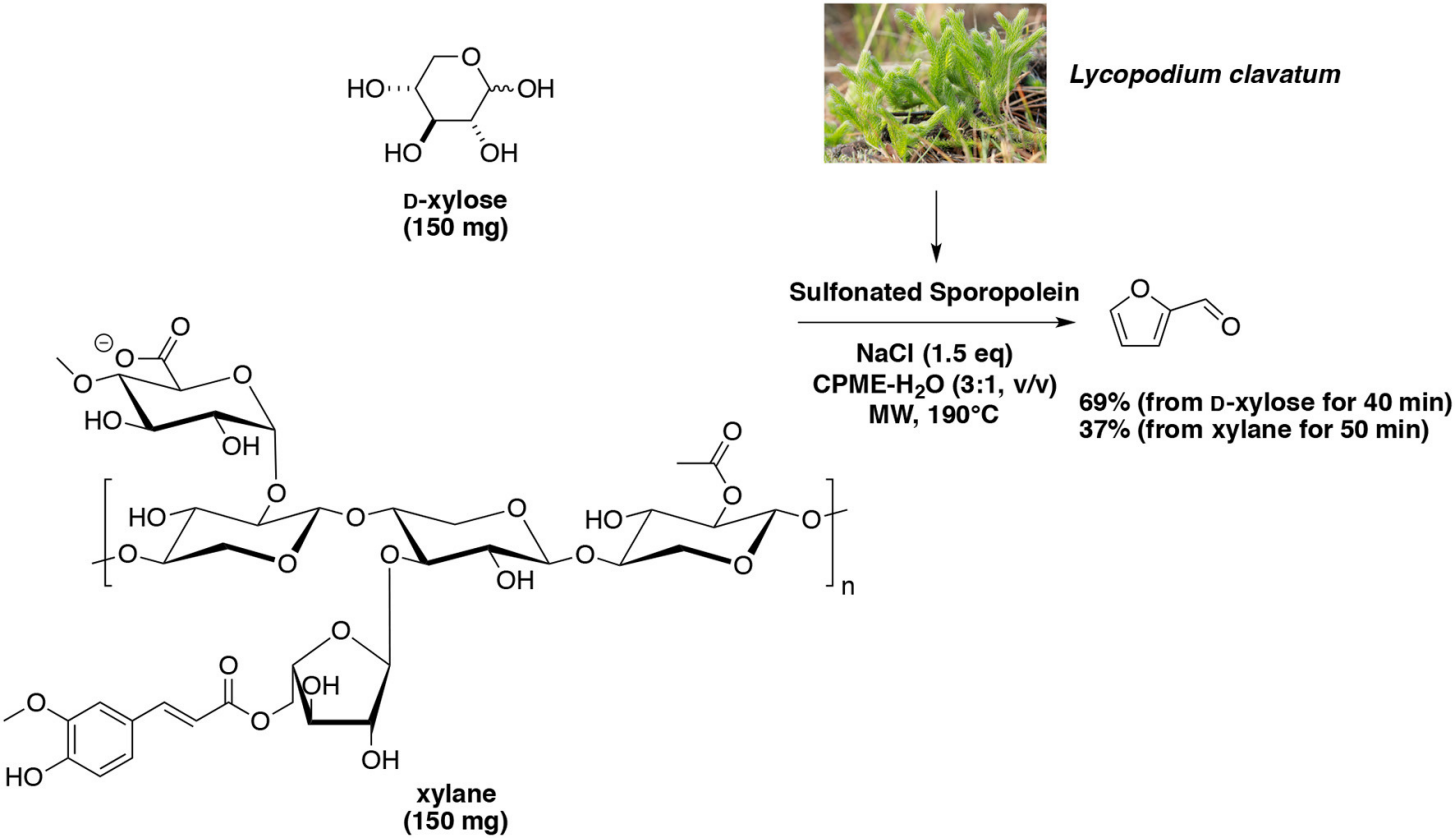
Microwave-assisted production of furfural from D-xylose in the presence of biobased sulfonated Sporopolenin in a mixture of H_2_O-CPME (Wang et al., 2017b).

Subsequently, a more stable and recyclable biobased catalyst sulfonated sporopollenin was successfully synthesized and tested for the microwave-assisted dehydration of D-xylose and xylan into furfural (Wang et al., 2017b). Under the same methodology developed by Len's group, sulfonated sporopollenin in the presence of NaCl furnished furfural in 69% yield from D-xylose at reaction temperature of 190°C and reaction duration of 40 min (Scheme 13). In our hands, when the catalytic system was recharged with carbohydrate and solvent, 10 catalyst recovery steps could be performed without loss of performance. Application to xylane at 190°C for 50 min furnished the aldehyde in 37% yield.

Using the same strategy, a new porous sulfonated triphenylamine was developed to dehydrate pentoses and hexoses in lactone-type organic solvents (Zhang et al., 2017a). When GVL was used as a solvent at reaction temperature of 175°C, furfural was obtained in 74% yield.

### Dehydration in presence of oxides

Different oxides derivated from Zr, Ti, and Nb have been studied for the production of furfural. Well-ordered mesoporous and super strong acidic sites catalysts ${\text{SO}}_{4}^{2-}$/ZrO_2_-Al_2_O_3_/SBA-15 were tested and the most efficient catalyst ${\text{SO}}_{4}^{2-}$/12% ZrO_2_-Al_2_O_3_/SBA-15 gave 99% of D-xylose conversion and 53% furfural yield when heating the 160°C during 4 h by Shi et al. (2011). It was found that the addition of Al to ${\text{SO}}_{4}^{2-}$/ZrO_2_/SBA-15 stabilized the tetragonal ZrO_2_ phase and enhanced the activity of the catalyst by increasing the number of acid sites. The reasons for catalyst deactivation and regeneration were examined. In spite of sulfur leaching, catalyst deactivation was related to the accumulation of humins, which might cover the surface of the catalyst, leading to the passivation of the acid site. Furthermore, deactivated catalyst can be completely regenerated with H_2_O_2_, but the furfural yield decreased by increasing the number of recycling tests, which was mainly caused by the presence of monoclinic ZrO_2_ that decreased the acid strength and acid concentration of the catalyst. Ultraviolet irradiation was tried with a screening of heterogeneous catalysts for the production of furfural from D-xylose (Li H.-L. et al., 2013; Li et al., 2014a), and from corncob (Li et al., 2014b). The optimized photocatalyst 1.0M-${\text{SO}}_{4}^{2-}$/TiO_2_-ZrO_2_/1.0wt%-La^3+^ gave 32% of D-xylose conversion and 216.6 μmol/g of furfural yield at 120°C for 4 h under ultraviolet irradiation: this is sharply higher than without catalytic process and absence of ultraviolet irradiation.

Some other scientists studied the simultaneous hydrolysis/dehydration of sugarcane bagasse, rice husk and corncob under hot compressed water in the presence of TiO_2_, ZrO_2_ and TiO_2_-ZrO_2_ at 200–400°C (Chareonlimkun et al., 2010). In all cases, TiO_2_ showed more efficiency in converting lignocellulosic biomass to furfural than ZrO_2_. It was found that catalysts prepared by (co-)precipitation method gained higher reactivity than those prepared by sol-gel and physical mixing methods. The suitable calcination temperature for TiO_2_ and ZrO_2_ was formed at 500°C. The use of mixed-oxide TiO_2_-ZrO_2_ (Chareonlimkun et al., 2010) offered the highest furfural yield (10%) from corncob at 300°C with less by-products selectivity when compared with TiO_2_ or ZrO_2_ at the same conditions. Five-time recycling of this oxides mixture, still ending up with a yield in furfural of 10%, indicated promising operational lifetime of this catalyst. Additionally, this catalyst has the bifunctionality for both acidity and basicity properties, which benefits to hydrolysis, dehydration and also isomerization processes, leading to high furfural production. The suitable calcination temperature for TiO_2_-ZrO_2_ was 600°C, which is higher than that for TiO_2_ and ZrO_2_.

Note that the introduction of sulfate ion and lanthanum can enhance the photocatalytic performance of metal oxides by increasing the acidic sites (Li et al., 2014b). Subsequently, this kind of catalyst was applied in the hydrothermal pretreatment of corncob into D-xylose and furfural (Li et al., 2014a). After optimizing the reaction parameters, the highest furfural yield (6.18 g of furfural for 100 g of corncob) could be obtained at 180°C for 120 min when the corncob-water ratio of was 10:100. From xylan, a furfural yield of 21% was achieved using Cr-LaCo_0.8_Cu_0.2_O_3_ (1.5%) as catalyst, under 10 h of conventional heating in the 1:600 weight ratio of xylan to water at 160°C (Li H.-L. et al., 2013). It is worth to notice that the amount of chromium in solid catalyst decreased from 1.26 to 0.38% after the first recycling run, which accordingly led to the reduction of furfural yield to 13%. Importantly, there is no effect on pentose formation.

An environmentally-friendly two-step process for furfural production starting from corncob (Deng et al., 2015). Hydrothermal pretreatment was firstly investigated in a temperature range from 160 to 190°C for reaction duration of 0–60 min, and then the resulted hydrolysate was catalyzed with ${\text{SO}}_{4}^{2-}$/SiO_2_-Al_2_O_3_/La^3+^ as a solid acid catalyst at 150°C for 2.5 h. The maximum yield of furfural achieved was 21% from the hydrolysates (total D-xylose yield 7.01 g/L) obtained at reaction temperature of 190°C for 60 min in the hydrothermal process, and no obvious decrease of furfural yield was detectable after four consecutive runs.

A mesoporous and amorphous nobium oxide was used to dehydrate D-xylose into furfural through a ketone-type intermediate called D-xylulose (Gupta et al., 2017b). Furfural selectivity (48%) was obtained in such environment when the reaction was carried out at 120°C, in sole water; however the yield increased in a water-toluene biphasic system (2:3, v/v) to reach 72% of selectivity. The catalyst activity was also compared to other solid catalyst such as titane oxide (TiO_2_).

### Dehydration in presence of sulfated oxides

Several bulk and ordered mesoporous silica-supported zirconia catalysts for the dehydration of D-xylose into furfural in a water-toluene solvent mixture at 160°C furnished furfural yields higher than 50% and a conversion higher than 90% (Dias et al., 2007). It should be noted that the initial catalytic activity correlated fairly well with the sulfur content. Unfortunately, sulfur leaching was observed compromising the reusability of the catalyst. The sulfated AZ-MCM-41 (prepared with 3.0 mmol ZrOCl_2_, 8 H_2_O and 0.1 mmol Al(NO_3_), 9 H_2_O) seems to be the most attractive catalyst for aqueous phase conversion of D-xylose, since it was the one offering the best inhibition to sulfur leaching and that in addition exhibited increasing activity and no significant loss of selectivity to furfural in three recycling runs, regardless of negative effect of Al addition on D-xylose conversion.

Recently, sulfonated carbonaceous materials revealed their potential for furfural production because of their high acidity, water tolerance and metal oxides having Lewis acidic sites. A carbonaceous heterogeneous catalyst, which combined the use of sulfonic acid (-SO_3_H) groups with Lewis acidic TiO_2_ in a one-pot synthesis method was prepared (Mazzotta et al., 2014). When testing this sulfated oxide for furfural production from D-xylose, the target chemical was obtained in 51% yield in DMSO as solvent at 140°C for 60 min or in water-MTHF biphasic system at 180°C for 30 min. Maximum furfural yield of 37% was also recorded in 5 min exposure at 180°C in DMA/LiCl-mediated reaction.

### Dehydration in presence of niobium-based oxides

Few works using niobium-based oxides were related for the dehydration of pentose into furfural. The activities of Lewis acid catalysts namely Nb_2_O_5_ supported on pyrogenic silica Cabosil (García-Sancho et al., 2014b) and Brönsted acid catalysts as Amberlyst 70 were studied (Agirrezabal-Telleria et al., 2012). Lewis acid sites gave a higher rate in the conversion of D-xylose into furfural than Brönsted acid sites did, because Lewis acid sites could isomerize D-xylose into D-xylulose, while *Amberlyst-70* with strong sulfonic acid sites presented a higher furfural selectivity. To improve furfural production, the use of N_2_ stripping methodology showed better extraction efficiency than water-toluene biphasic system which benefits to environmental and products separation concerns. Nb_2_O_5_ catalyst was prepared by a neutral templating route (García-Sancho et al., 2014a). The catalytic behavior was compared with that of a commercial Nb_2_O_5_. Higher than 90% of D-xylose conversion and 54% of furfural yield was attained at a temperature of 170°C after 90 min of exposure in presence of a D-xylose-catalyst (3:1, wt/wt). It is noteworthy that 41% of furfural can be achieved at 150°C for the same reaction time. In addition, D-xylose conversion in the non-catalyzed process was practically negligible, and commercial Nb_2_O_5_ only gave low D-xylose conversion (22%) and very low furfural yield (3%). It was demonstrated that no significant niobium leaching has occurred, the catalytic activity reduced could be attributed to the presence of a large amount of carbonaceous deposits on the catalyst surface. Silica-zirconia supported niobium catalysts were prepared by impregnation (Nb/SZi) or sol-gel (Nb/SZsg) for comparison of their catalytic effect to pure niobic acid (NBO), in the context of D-xylose dehydration in green solvents: water, water-isopropanol, water-GVL, and water-CPME (Molina et al., 2015). In all the solvents, NBO was found more active than the other supported niobium catalysts. All the supported niobium catalysts had got the same activity and showed the highest D-xylose conversion and furfural yield in presence of GVL and CPME. During the recycling and continuous catalytic tests, in spite of the deactivating trend of D-xylose conversion, furfural yield is more stable in such conditions. The niobia supported catalysts promoted furfural production in the range of 45–50% yield and almost 80% selectivity after 7 runs at 180°C for 4 h. These catalysts have lower activity than bulk niobia but higher stability. Note that furfural yield could reach around 60% by increasing reaction time to 4 h at 180°C or reaction temperature to 190°C for more than 2 h catalyzed with NBO.

### Dehydration in presence of phosphates

Different phosphates associated with Vanadium, Niobium and Tantanum were used as catalysts for the dehydration of pentose derivatives. The orthorhombic vanadyl pyrophosphate (VO)_2_P_2_O_7_, prepared by calcination of VOHPO_4_, 0.5H_2_O at 550°C for 2 h, exhibited superior catalytic performance amongst the investigated materials. In this work furfural yield (53%) in consecutive batch runs at 170°C for 4 h was reported (Sádaba et al., 2011). Interestingly, this heterogeneous catalyst actually behaved as a source of very active water-soluble species, which are responsible for the observed catalytic activity. A series of zirconium phosphate catalysts have been synthesized (Cheng et al., 2013). The calcined mesostructural zirconium phosphate ZrP-HT-Am-C exhibited inspiring catalytic performance. At 170°C, D-xylose conversion (96%) and furfural yield (52%) were measured in aqueous-phase after 2 h. The reusability of the catalyst as evaluated from 3 runs seemed fairly good, however conditioned by thermal treatment in air at 500°C for 4 h between runs. Association of niobium and phosphate was done and the corresponding nobium phosphate (NbP) was tested for furfural production (Bernal et al., 2015). Nb^5+^ ions were supported to act as Lewis exposed sites, showing medium strength acidity. Their previous study showed that NbP recovered catalyst gave furfural in 43% yield and D-xylose conversion in 82% at 160°C for 30 min in water (Bernal et al., 2014). After three consecutive catalytic runs, furfural yield and D-xylose conversion slightly decreased to 36 and 70%, respectively. When corn stover was tried as feedstock, 23% of furfural yield was achieved, only. Eventually, another tentative use of phosphates as heterogeneous catalyst for biomass dehydration was targeting a synergistic effect of tantalum (Li X.-L. et al., 2015). In this regard, different mesoporous tantalum phosphates (TaOPO_4_-m) with various P/Ta molar ratios were prepared and tested for evaluating their respective catalytic activities to convert D-xylose into furfural (Xing et al., 2016). A high Bronsted to Lewis acid site ratio is required to enhance the furfural selectivity. The best candidates afforded a furfural selectivity of 72%.

### Dehydration in presence of silicates

The main silicates used for the dehydration of biomass into furfural have been mesoporous silicates *MCM-41* and *SBA-15* and derivatives having sulfonated group or metals. They were tried for the dehydration of pentose into furfural. Among the different silicates, *H-MCM-41* gave the highest furfural yield was obtained in the context of unusual C6 sugar levoglucosan treatment at 300°C. Unfortunately, catalysts were deactivated due to coke formation (Käldström et al., 2010). The conversion of D-xylose into furfural with mesoporous molecular sieve *MCM-41* as catalyst in presence of NaCl and butanol as the extraction solvent with treatment at 170°C for 2 h gave a production of furfural in 48% yield (Zhang et al., 2012). In three consecutive runs, its value decreased slightly, which can be due to inefficient removal of by-products adsorbed on the catalyst surface after each run. It was noteworthy that the same process without addition of NaCl gave only the aldehyde in 39% yield.

Metal-containing silicates (Nb, Al) were also tested and compared. In this context, the microporous and mesoporous niobium silicates (*AM-11* and *MCM-41*) as solid acid catalysts were studied for the dehydration of D-xylose in a water-toluene solvent mixture (Dias et al., 2006a). The proton form of *AM-11* (*H-AM-11*) showed a proven reusability and gave the highest furfural yield (50%) and a D-xylose conversion (89%) at 160°C for 6 h after 3 runs. For fresh catalysts, their activities followed the order: *H-AM-11* (46% at 85% conversion) > *ex H-AM-11* (*H-AM-11* after ions-exchange, 39% at 85% conversion) ≈ *HY5* (the protonic form of Y-zeolite, with Si/Al = 5, 39% at 94% conversion) ≈ *MCM-41* (*Nb50-MCM-41, ex Nb50-MCM-41, Nb25-MCM-41* and *ex Nb25-MCM-41*, ca. 39% at 92~99% conversion) > *Na,H-AM-11*(31% at 77% conversion) > mordenite (with Si/Al = 6, 28% at 79% conversion). Developing the same idea, the effect of surface acidity on the dehydration of D-xylose was examined using SiO_2_-Al_2_O_3_ catalysts with varying alumina contents by You et al. (2014). D-xylose conversion in water as solvent at 140°C for 4 h increased more than 9 fold with the increase of alumina content from 0 to 1. Simultaneously, humins yield reached 45% with Al_2_O_3_ whereas none was formed with SiO_2_. It was considered that Lewis acid sites significantly affected D-xylose conversion and humins formation. The best furfural yield of ca. 27% was obtained with SiO_2_-Al_2_O_3_ (0.6) while SiO_2_-Al_2_O_3_ (0.1) provided the highest furfural selectivity (over 60%). The highest yields of D-lyxose and D-xylulose were attained over SiO_2_-Al_2_O_3_ (0.4) and SiO_2_-Al_2_O_3_ (0.8).

Silicates with sulfonated groups have been tested for the model reaction. Two kinds of sulfonic *MCM-41* catalysts: the propyl sulfonic acid catalyst (*PrSO*_3_*H-MCM-41*) and the methyl propyl sulfonic acid catalyst (*MPrSO*_3_*H-MCM-41*) have been prepared (Kaiprommarat et al., 2016). After testing their activities for furfural production from D-xylose in water-toluene biphasic system at 155°C for 2 h, it was found that *PrSO*_3_*H-MCM-41* was quite efficient and gave 96% of furfural selectivity and 92% of D-xylose conversion compared to 26% and 99% for *MCM-41*. The authors explained that the preparation of *MPrSO*_3_*H-MCM-41* has a significant effect on their acidic densities and pore diameters, which further affect furfural yield and D-xylose conversion. *MPrSO*_3_*H-MCM-41* prepared by co-condensation method using dodecyltrimethylammonium bromide as a surfactant template and aged at room temperature gave the smallest pore diameter (3.4 nm). The modification of the catalyst permitted to furnish the highest furfural yield (93%) and selectivity (98%). The reusability of the *MPrSO*_3_*H-MCM-41* needs to be challenged as furfural yield rapidly decreased below 50% as soon as recycled for the first time. Mesoporous shell silica bead (*MSHS*) served as support and modified with sulfonic acid (*MSHS-SO*_3_*H*) and aluminum (*MSHS-Al*) were prepared (Jeong et al., 2011). Their catalytic performance in the dehydration reaction of D-xylose into furfural under water phase was investigated. *MSHS-Al* gave a higher D-xylose conversion than *MSHS-SO*_3_*H* (45 vs. 32%), but showed a lower furfural selectivity (35 vs. 57%). Moreover, *MSHS-Al* isomerized D-xylose to D-lyxose at a concentration of 14%. When compared to general mesoporous catalysts *MCM-41-SO*_3_*H* and *HMS-SO*_3_*H, MSHS-SO*_3_*H* is more efficient than *HMS-SO*_3_*H* (18 vs. 13% for furfural yield at 170°C for 1 h), but less efficient than *MCM-41-SO*_3_*H* (24% in furfural yield) which attribute to its almost three times the number of sulfonic acid groups compared to *MSHS-SO*_3_*H*. In addition, *MSHS-SO*_3_*H* showed a better hydrothermal stability than *MCM-41-SO*_3_*H*.

### Dehydration in presence of zeolites

Different zeolites have been prepared and tested for the dehydration of carbohydrate derivatives into furfural. Zeolite catalysts [zeolite SM-25, mordenite 13 (Si/Al = 13), mordenite 20, faujasite 13] acidified with H_3_PO_4_ or H_2_SO_4_ were tested for D-xylose dehydration to furfural in a continuous two-liquid-phase (water-toluene) plug-flow reactor (Lessard et al., 2010). The optimal conditions were determined: powdered mordenite (H^+^) 13 as the catalyst, 12% w/w D-xylose solution, a reactor temperature of 260°C, a pressure of 55 atm, a toluene/D-xylose aqueous solution volumic ratio of 2, and a residence time of 3 min. For the first cycle, the furfural molar yield reached 98% with a conversion rate of 99%, and the second pass gave a furfural yield of 90% associated with the same conversion rate. This indicated that the regeneration of the mordenite cannot recover its original activity.

A series of solid catalysts were tested for selectively converting hemicellulose from crop waste into C5 sugars and furfural (Sahu and Dhepe, 2012). The *HUSY* (Si/Al = 15) catalyst showed the highest activity to convert hemicellulose (>90% conversion) at 170°C within 3 h in the presence of water, followed by *H-Beta* zeolite (Si/Al = 19), *HMOR* zeolite (Si/Al = 10), and K10 clay. However, herein, less than 12% of furfural formed. Other catalysts: γ-Al_2_O_3_, Nb_2_O_5_, and Al-containing mesoporous silica exhibit less activity. The application of a biphasic solvent mixture furnished higher furfural yield. For example, 54-56% of furfural yield was obtained in water-toluene or water-MIBK or water-xylene biphasic system catalyzed with *HUSY* (Si/Al = 15) at 170°C for 6 h. Zeolites seemed to be hydrolytically stable in the reaction mixture and mineral impurities such as Na and K in biomass may be responsible for the reduction in the activity of the catalysts. *ZSM-5* zeolite catalyzed the furfural production from aqueous hemicelluloses solution [which contains D-xylose (164 g/L), D-glucose (11 g/L) and arabinose (4.5 g/L)] (Gao et al., 2014). The effects of reaction temperature, time, catalyst loading, organic solvents and inorganic salts or metal oxides addition were thoroughly investigated. The maximum furfural yield of 82% and the D-xylose conversion of 97% were achieved at 190°C in the presence of *ZSM-5* (1.0 g), NaCl (1.05 g) and organic solvent-to-aqueous phase ratio of 30:15 (v/v) for 3 h. Applying these conditions for conversion of pure D-xylose solution decreased the yield of furfural (51%) which was attributed to easier occurrence of excessive hydrolysis of D-xylose and condensation reactions. Besides, *ZSM-5* has a relative stability and can be reused at least five times with the furfural yield remaining over 67%.

*HZSM-5* zeolite was used to improve the production of monoaromatic hydrocarbons, such as benzene, toluene, xylene and ethylbenzene. In fact, usually there is only little amount of furfural formed with this catalyst (less than 0.59 wt% with respect to dry basis sugar maple) (Mante et al., 2014). The same catalyst *HZSM-5* helped however to produce furfural from steam explosion liquor of rice straw (which contains mainly D-xylose oligomers 2.27 kg/m^3^ in 3.61 kg/m^3^ total sugar) (Chen et al., 2015). The maximum furfural yield was 310 g/kg under the optimum conditions: *HZSM-5* addition 60 g/kg sugar, reaction temperature 160°C, extraction steam flow rate 2.5 cm^3^/min and total sugar concentration 61.4 kg/m^3^. It was worth noting that polymerization inhibitor 4-methoxyphenol and tert-butylcatechol were added into the reaction system to improve furfural yield. It was found that 4-methoxyphenol was more efficient than tert-butylcatechol at the same additive amount, especially with 15 g/kg 4-methoxyphenol addition increased furfural yield to 375 g/kg, which increase by 21% compared with that without polymerization inhibitor. *HZSM-5* catalysts could be reused for 3 runs and the catalytic activity recovered 88% after regeneration through calcinations.

D-Xylose dehydration activity of arenesulfonic *SBA-15* catalysts synthesized at high aging-temperature (180°C) revealed that the catalyst was more selective and hydrothermally stable (Agirrezabal-Telleria et al., 2014a). For example, *SBA-15* modified with 0.2 mole ratio of 2-(4-chlorosulfonylphenyl) ethyltrimethoxysilane (A180-0.2) gave furfural in 82% yield with a conversion of 98% at 160°C in water-toluene biphasic system for 20 h. Modification of the catalyst with 0.3 organosiloxane molar loading led to furfural in 86% yield with a conversion of 99%. Amberlyst-70 was compared with arenesulfonic *SBA-15* catalysts, which presented obviously lower furfural selectivity. Moreover, regenerated A180-0.2 by using a thermal treatment at 290°C gave furfural in 75% yield and a conversion of 88% after three consecutive runs. The catalysts aged at lower temperature showed important deactivation rates.

Microporous silico aluminophosphates *SAPO-5, SAPO-11* and *SAPO-40* were tested as solid acid catalysts for the dehydration of D-xylose into furfural under water-toluene biphasic system at 170°C (Lima et al., 2010). Furfural yields in 4 h using *SAPO-11* (34–38%) are comparable with that for *HMOR* zeolite with Si/Al^*^6 (34%). under similar reaction conditions, while *SAPO-5* and *SAPO-40* gave less than 25% of furfural yield. Complete D-xylose conversion is reached within 16–24 h, with furfural yields in these conditions of up to 65%. No decrease of Si, P, or Al contents and furfural yield were observed in all catalysts for three consecutive runs. In another work, *SAPO-44* revealed superior efficiency to *SAPO-5, SAPO-11*, and *SAPO-46* for one-pot conversion of hemicellulose into furfural since it had higher acid amount and surface area (Bhaumik and Dhepe, 2013). With respect to HMOR (Si/Al = 10), even if it has an equal total acid amount of 1.2 mmol/g and a higher surface area than *SAPO-44*, an inferior activity was observed because of its weaker hydrophilic nature. SAPO-44 could give a furfural yield of 63% with 88% of mass balance at 170°C within 8 h and no loss of catalytic activity was observed after 8 cycles. Later, more hemicellulose type of feedstocks were tested (Bhaumik and Dhepe, 2014), and extraordinarily high yields of furfural through catalysis making use of *SAPO-44* were obtained (about 86-93% from bagasse, rice husk and wheat straw) when these biomasses were treated at 170°C in a water-toluene biphasic system. IN this context of use, *SAPO-44* could also keep consistent activity. Subsequently, adequate engineering of SAPO-44 catalysts for efficient synthesis of furfural from xylan was investigated (Bhaumik and Dhepe, 2015). It was found that SAPO-44 having 1.0 mole of Si content is the best catalyst for the xylan-D-xylose conversion to furfural. The use of a biphasic ratio of 1:2 (v/v) showed the highest amount of furfural (82%) production from xylan when processed at 170°C for 10 h of reaction time. Other kinds of catalysts such as *H*β (Si/Al = 19), *HMOR* (Si/Al = 10), *HUSY* (Si/Al = 15), were also compared with *SAPO-44*. Recently, small pore zeolites *SAPO-34* and *SAPO-56* were considered and tested for furfural production from D-xylose and switchgrass in water-GVL monophasic system (Bruce et al., 2016). *ZSM-5, Amberlyst-70* and H_2_SO_4_ led to a better furfural yields (70, 63, 67% from D-xylose respectively), but leaching studies indicated that these good results were attributed to homogeneous catalysis by the acid sites that leached from the catalysts. The commercial *SAPO-34* catalyst gave a moderate furfural yield of 40% from D-xylose and 31% from switchgrass, and showed a good recyclability. At the light of their emerging use, these small pore zeolites may be rationally designed to increase the yield from biomass reactions.

The use of a chabazite-type zeolite prepared from the chemical transformation of a faujasite-type natural one was studied (Yoshida et al., 2017). The hemicellulose contained in ball-milled pretreated bamboo powder was directly transferred into furfural when it was stirred in a water-toluene biphasic system under conventional heating. From this hemicellulose processed in this biphasic system at 170°C for 10 h, furfural was finally produced in 55% yield.

A special zeolite displaying iron, tin and zirconium sites was synthesized from H-β type material by means of an ion-exchange procedure (Zhang et al., 2017b). The catalyst prepared in this way holds both Bronsted and Lewis acid sites. Surprisingly, by using the more effective Sn-beta, D-glucose became the substrate to produce furfural when heated at 180°C. In a water-GVL biphasic system, furfural was obtained in 69 % yield.

## Synthesis of furfural from sugars and polysaccharides using supported catalysts

Mesoporous silica-supported 12-tungstophosphoric acid (PW) catalysts showed a significant effect on the catalytic performances in biomass conversion to furfural relating to several variables, such as the catalyst preparation method, type of support, PW loading, and the reaction conditions (Dias et al., 2006b). In water-toluene biphasic system, catalysts prepared in 1-butanol reveal better reusability through recycling runs than when prepared in water. Besides, higher PW loadings and temperatures both led to higher furfural yield. The furfural yields and catalyst activity resilience were higher in DMSO than in water-toluene. Furfural was produced at 140°C for 4 h in DMSO in 52% yield when the catalyst was prepared in 1-butanol with 34 wt% of PW supported on medium-pore micelle-templated silica. However, the best catalytic stability performance versus use was obtained in DMSO using either the 15 wt% PW inorganic composites, or PW immobilized in aminopropyl-functionalized silicas. Generally, the catalyst deactivation was due to PW leaching and catalyst surface passivation.

Later, they fixed cesium salts of 12-tungstophosphoric acid on medium-pore MCM-41 (3.7 nm) or large-pore (9.6 nm) micelle-templated silicas and investigated their catalytic performance for D-xylose dehydration to furfural in water-toluene and DMSO solvent systems (Dias et al., 2006c). In this work, similar conclusions have been observed. The initial catalytic activities decreased in the order silica-supported CsPW > Cs_2.5_PW > Cs_2.0_PW > HPW. Increasing the CsPW loading from 15 to 34 wt% or using a support with a larger pore diameter, nearly doubled furfural yields.

A series of MCM-41-supported niobium-oxide catalysts were also tested. In general, the catalytic activity of this family of catalysts is related to the presence of niobium species over the silica support. The catalytic activity improves with the increase in the niobium-oxide content (García-Sancho et al., 2013). However, the catalyst with 16% of Nb_2_O_5_ loading (MCM-Nb16) reveals more efficient than that with 33% of Nb_2_O_5_ loading which resulted in closure of the pores and partial destruction of the mesoporous framework. *MCM-Nb16* has a remarkable ability, including reusability for furfural selectivity, whatever the increase in conversion efficiency or the reaction temperature. Moreover, in addition of 0.5 g NaCl/g aqueous solution, furfural yield significantly increased from 36 to 60% at 170°C for 180 min in water-toluene biphasic system. Indeed, three consecutive runs did not reveal any drop in catalytic activity with the recovered catalyst. Subsequently, the study of niobium oxide incorporated on different supports indicated that the textural properties of the supports and the total acidity both played significant roles in D-xylose dehydration and furfural selectivity (García-Sancho et al., 2014b). γ-Al_2_O_3_ showed the highest D-xylose dehydration activity but by contrast low furfural selectivity. This was analyzed as a consequence of its higher than Brønsted Lewis acidity, which might favor side reactions on the catalyst surface. Commercial fumed silica supported catalyst presented larger pore sizes, favoring the diffusivity of D-xylose and consequently the dehydration activity. Whereas *SBA-15* and *MCM-41*showed higher acid site densities, their intermediate micro-mesoporous structure could provide higher D-xylose dehydration rates to furfural. *SBA-15* with 12 wt% niobium oxides loading showed the highest amount of acid sites than catalyst variants containing 4 wt% and 20 wt% niobium oxides loadings. This catalyst gave a D-xylose conversion of 85% and furfural selectivity of 93% in reported process conditions (160°C for 24 h in water-toluene mixture). Moreover, the regenerated catalyst showed similar furfural selectivity with 4% of D-xylose conversion decrease.

Hydrothermal pretreatment of corncob with microwave-assisted irradiation has been systematically studied. Their subsequent hydrolysates with the maximum D-xylose content (160°C, 90 min), the maximum xylobiose content (180°C, 15 min), and the maximum total D-xylose content in monosaccharide and oligosaccharides (DP ≤ 6) (160°C, 60 min) were further processed to produce furfural using tin-loaded montmorillonite (Sn-MMT) as the catalyst in the 2-sec-butylphenol/NaCl-DMSO system, respectively (Li H. et al., 2015). The highest furfural yield (58%) was obtained from the hydrolysates with the maximum D-xylose content, whilst the lowest furfural yield (less than 10%) was issued from processing the hydrolysate with the maximum xylobiose content. This result indicated that the production of furfural has a direct connection with the monomeric pentose. Controlled experiments with pure D-xylose solution showed lower furfural yield than that from the hydrolysates with the same total D-xylose amount in monosaccharide and oligosaccharides, which may be related to the slow release of pentose monomers from the oligomers that can impede the formation of humins.

A silica supported poly(styrene sulfonic acid) was prepared in another recent study. The silica particles were first functionalized by means of aminopropyltriethylsilane and showed primary amine moieties on its surface to attach the polymer through electrostatic interactions (Campo-Molina et al., 2017). The catalysts were tested for their capacity to dehydrate D-xylose in the organic solvents. The best performance was obtained using a 10 wt% D-xylose aqueous solution in water-CPME biphasic system. When the medium was heated at 180°C for 60 min, the furfural yield eventually reached a value of 57%.

A series of functional IL supported silica nanoparticles with different acidity (ILs/SiO2) have been prepared by covalent bonds using non-toxic ethanol as solvent (Xu H. et al., 2015). With respect to furfural production from D-xylose, the catalytic performance followed the order: IL/SiO_2_ < IL-SO_3_H/SiO_2_ < IL-HSO_4_/SiO_2_ < IL-SO_3_H-HSO_4_/SiO_2_, and furfural yield increased from 31% to 50% at similar D-xylose conversion efficiency of about 94-96%. In the catalyst IL-SO_3_H-HSO_4_/SiO_2_, the strong acid sites from SO_3_H and ${\text{HSO}}_{4}^{-}$ have played the roles of active centers. As for IL/SiO_2_, 31% of furfural yield was attributed to positive effect of chlorine anion from the IL.

## Synthesis of furfural from sugars and polysaccharides using other catalysts

Other catalysts were tested in that context for their activity regarding the production of furfural. Vanadium (10 wt%) contained *H-MCM-41* catalysts showed the highest catalytic activity for the production of furanic compounds (e.g., the most abundant furanic compound was furfural) during the ex situ catalytic pyrolysis of cellulose, levoglucosan and xylan (Kim B.-S. et al., 2016). It was found that furanic compounds were mainly derived from levoglucosan over weak acid sites of vanadium contained H-MCM-41.

The catalytic activity of functionalized partially hydroxylated MgF_2_ catalysts for D-xylose dehydration into furfural was reported (Agirrezabal-Telleria et al., 2013b). Partially hydroxylated MgF_2_ which contains Lewis and Brønsted sites, was further modified with perfluorosulfonic or methanefluorosulfonic acids. The former sulfonic precursor showed higher selectivity for furfural than the latter, primarily due to a lower sulfur atom incorporation and also as a consequence of the reduction in furfural resinification reaction rates. Well-optimized Lewis/Brønsted ratios catalyst synthesized by one-step grafting technique could give a maximum furfural selectivity of 90% at 160°C in a water-toluene biphasic system. Another part of their research investigated D-xylose conversion to furfural with partially hydroxylated MgF_2_ catalysts synthesized using different HF concentrations (Agirrezabal-Telleria et al., 2014b). *MgF*_2_-*71* (synthesized with 71 wt% HF) gave 86% of furfural selectivity with 94% of D-xylose conversion in water-toluene and subsequently, a furfural selectivity of 87% could be achieved using N_2_-stripping. The catalyst *MgF*_2_-*40*, which is rich in Lewis acid-sites promoted the D-xylose conversion rather than furfural selectivity, and *MgF*_2_-*87*, which has lower Lewis acid-sites content gave opposite trends. It was found that the addition of D-glucose as a co-carbohydrate decreased furfural selectivity in all case. The change of reaction mechanism depended on the catalysts containing different Lewis/Brønsted ratios.

Furfural production using solid acid catalysts in GVL was studied in the presence of γ-Al_2_O_3_, *Sn-SBA-15* and Sn-beta, which contain only Lewis acid-sites (Gürbüz et al., 2013). In their work, furfural was obtained in low yields (<40%). Sulfonic acid functionalized catalysts *Amberlyst-70, Nafion SAC-13*, sulfonated carbon, and propylsulfonic acid functionalized *SBA-15*, zeolites (*H-ZSM-5, H-mordenite*, and *H-beta*), sulfated inorganic metal oxides (sulfated zirconia), and even mineral acid H_2_SO_4_ have been tested. The effect of water content in GVL was investigated since it is known water has significant influence on furfural degradation reactions. Under the best conditions, 81% furfural and 4% formic acid were obtained in GVL with 10% water using *H-mordenite* as catalyst, and no decrease in furfural yield after five cycles, which is more stable than *Amberlyst-70* and propylsulfonic acid functionalized *SBA-15*. Interestingly, the main product of D-glucose conversion using zeolite catalysts and GVL as the solvent is furfural with a yield superior to 30%

A mechano-catalytical strategy aiming to simultaneously release hemicelluloses from the corncob cells and depolymerize the polysaccharide into its pentose subunits in presence of a solid acid catalyst (${\text{SO}}_{4}^{2-}$/ SiO_2_-Al_2_O_3_/ La^3+^) was published (Li et al., 2016). Herein, ball-milling treatment played an important role in the decomposition of the crop material. During the sonication step, the acid catalyst activated the conversion of the hemicelluloses content into furfural. Finally, furfural yield of 83% was obtained at 190°C for 30 min from the pretreated waste material.

Currently metal organic frameworks (MOFs) are emerging as promising new materials according to their catalytic properties favoring various reactions. A good example is the use of Zn_2_(Bim)_4_ as an organic filter embedded in polymethylphenylsiloxane (PMPS) (Jin et al., 2016). Both are components of a porous composite membrane potent for the vapor permeation and isolation of produced furfural. After recovery of the product, 41% yield in furfural was obtained when operated at 140°C in aqueous solution.

## Mechanism for the synthesis of furfural

**Scheme 14:**
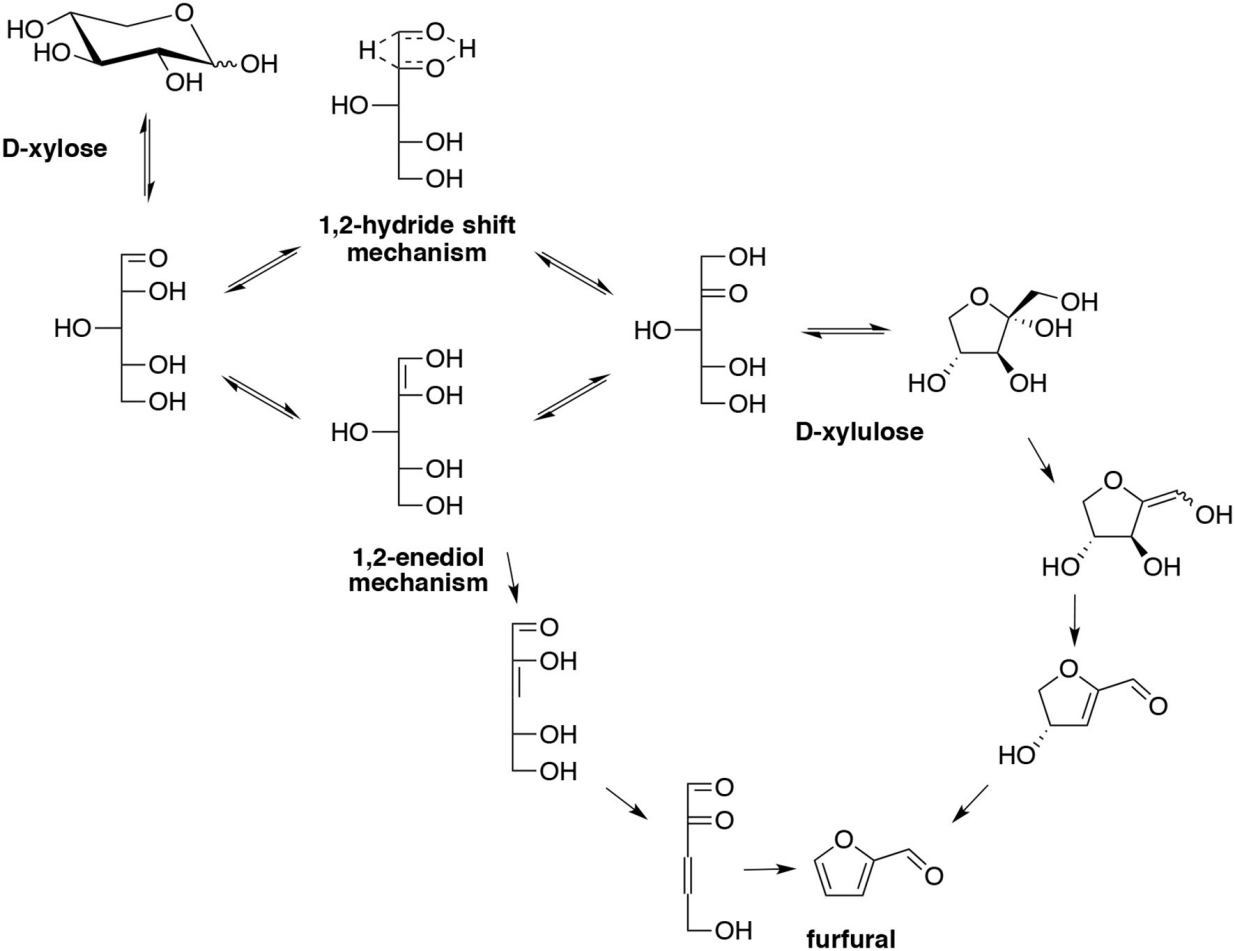
Plausible mechanism of D-xylose reaction to furfural in acidic media (Danon et al., 2014).

Different mechanisms of the formation of furfural from D-xylose have been identified and described in various researches reported in the literature. Among them, different keys steps have been explored using cyclic or acyclic pathways: 1,2-enolization, β-elimination, isomerization *via* 1,2-hybride shift. A plausible mechanism was proposed based on mechanistic and kinetic aspects in aqueous media employing homogeneous catalysis (Danon et al., 2014). Starting from acyclic D-xylose, the isomerization *via* 1,2-hydride shift or 1,2-enediol mechanism afforded the corresponding ketose, which was the key intermediate for assuming the cyclic pathway in the mechanism. By contrast, the 1,2-enediol derivative could supply the 2,3-unsaturated aldehyde as a key intermediate if the assumption of the acyclic pathway is made (Scheme 14). Such uncertainties are reflected in the contradictory kinetic models exploited and kinetic data presented in the literature, which still prevent scientists today from a common and coherent interpretation.

## Safety considerations

Furfural is likely to be the first bio-based industrial chemical ever produced, by Quaker Oats Company since 1920. Furfural has been identified as one of the 30 building block chemicals by the US Department of Energy, as this is one of the widely studied chemicals since nineteenth century and has been referred by many well recognized handbooks and data sheets (Werpy and Petersen, 2004; Peleteiro et al., 2016a). Owing to its physico-chemical properties, production methodologies and its vast industrial applications, it is important to consider potential risks of furfural during its entire life cycle (feedstock storage, production, transportation, furfural storage, end user application, and disposal). Industrial production of furfural is generally dealt with homogeneous or heterogeneous catalytic conversions as described in detail in this review. Homogeneous catalysts can lead to serious operational, safety and environmental issues during the production of furfural in several ways. It is very difficult to separate the mineral acids for recycling, which may lead to some product contamination. These acid catalysts are highly corrosive and might lead to equipment damage and process breakdown and they are toxic and may cause problems during disposal. Heterogeneous catalysis on the other hand may also trigger some hazards during use or recycling (Lamminpää et al., 2012; Melero et al., 2012; Ventura et al., 2016). In the case of solid acid catalysts, depending on the hydrophilic or hydrophobic properties of the chosen (solid acid) catalyst, there is a tendency of severe poisoning of acid sites by water, which loses the catalytic activities in aqueous solutions (Okuhara, 2002).

Based on its physical or chemical properties, furfural is qualified as a dangerous substance and listed under various internationally derived hazardous-material classifications such as UN's GHS [CLP in the EU (Regulation (EC) N° 1907/2006 and 453/2010)] (United Nations, 2015), TDG Regulations based on the UN's “Orange Book” etc. GHS labeling categorizes furfural as toxic by various routes (United Nations, 2009, 2015). Whereas, according to US NFPA 704 Hazard ranking “Standard System for the Identification of the Hazards of Material for Emergency Response,” furfural is qualified as instable (hazard rating level 1), flammable (hazard rating level 2) and toxic (hazard rating level 3) (CAMEO Chemicals, 2016^1^).

[6]Furfural (flash point (FP) 60°C) clearly has flammability properties and forms air/vapor mixtures above 60°C and these vapor-air mixtures are explosive within known flammable limits (2.1–19.3%) (Urben and Pitt, 2007). Prior to the amendment in CLP regulations, i.e., according to the Regulation (EC) n0 440/2008, furfural with FP 60°C was considered as non-flammable (as a flash point of 55°C was the recognized upper limit of flammable liquids). Due to the modifications in CLP regulations with new (expanded) FP limits (the new upper limit designing “flammable substances” is by now related to FP ≤ 60°C), furfural should have fallen into 3rd category of flammable substances due to most quoted value of furfural flash point (60°C), while it has not been the case in practice, since the risk evaluation by ECHA has retained a flash point of 61.7°C just over the new flammability limit of category 3 of CLP flammable liquids (Chemical Substances Bureau, 2008^2^). It is noteworthy that, actual physico-chemical properties (particularly the flammability hazard) and its associated risks remain the same irrespective of any classification change.

Early research quotes that furfural has a tendency to impart extreme corrosiveness toward carbon steel equipment (Scarth, 1947). Therefore, in-spite of its property of selective absorption of unsaturated hydrocarbons, furfural may not be a suitable solvent in extractive distillation process. Laboratory tests quote that, the rate of corrosion of furfural on carbon steel is over 1.0 mm/year which can cause serious corrosion (Sandvik Material Technology, 2016). In many aspects, corrosion management keeps a complex issue, since the degree of corrosion may also depend on the types of materials and chemicals associated with it, and the type of application.

On the other hand, furfural (as an organic compound) by contrast tends to get adsorbed on the metal-solution interface, which in turn tends to reduce corrosion of metal surface. Some electrochemical tests have outlined some corrosion inhibition effect on low carbon steel when furfural is in ethanol solutions, with an effective concentration of 0.1 mM of furfural (Goncalves and de Olivera, 1992). Moreover, some investigations proved that aliphatic furfural hydrazine derivatives act as corrosion inhibitors of iron in nitric acid (Mohamed et al., 1989). Results from the study conducted confirmed a corrosion inhibition effect of furfural in the presence of HRWR (high range water reducer) superplasticizers (Al-Hubboubi et al., 2012).

Dealing with hazards to health, furfural is qualified as a CMR carcinogen (cat.2, H351) in CLP (Regulation (EC) No 790/2009) and has found to impart toxic effects of human body through inhalation (cat.3, H301), oral consumption (cat.3, H331), dermal absorption (cat.4, H312) and eye irritant (cat.2, H319). In the recent amendment of CLP, furfural is additionally recognized as a skin irritant (cat.2, H315). Although a few animal toxicity tests (with an emphasis on genotoxic, mutagenic and carcinogenic properties) have confirmed some conventional toxicity rating, apparently, no significant threat to human health has actually been recognized from medical observations so far as reported in the literature. As the effect of human exposure of furfural is still under debate, any extrapolation of available animal test results to humans will be highly questionable (Scientific Committee on Consumer Safety, 2012; Abbott, 2016).

Chemical accidents involving furfural may not only occur during manufacture and use, but also during handling, transportation, storage and disposal stages. Although there are no major furfural accidents reported in the recent years, some case studies summarized hereafter demand vigilance while handling this chemical. Several cases of spontaneous ignition were observed when the fine coke particles (containing furfuryl alcohol derived resin) were exposed to air after their removal from the filter strainers in a petroleum refinery furfural extraction unit. In a regenerative furfural distillation while distilling furfural under nitrogen, furfural residues agglomerated in the boiler. Despite tentative inserting, fire broke due to air entering the boiler from bottom valve due to scrapping of walls because of too long operation, unidentified substances in the solvent forming peroxides, poor waste acceptance procedure etc. Seal of the connection at receiving end broke in a petroleum refining unit due to the corrosion of metal joints resulting in furfural splash on the employees. Rejection of 15 ton of furfural solvent (via cooling water) from a petroleum refinery due to a leak in the heat exchanger of a unit separating aromatics distillate led to the pollution of the Tancarville channel covering around 2 km, resulting in 400 kg of dead fish. A crash between 2 trucks during the transportation of furfural and acetic acid resulted in immediate ignition of trucks and the death of truck drivers. A leak from undetermined number of drums containing furfural led to emergency management difficulties due to lack of available containment equipment to handle flammable liquids; inspecting staff with inadequate knowledge thus leading to wrong methods of handling flammable liquids (ARIA, 2016). Reported case studies clearly depict issues relating to flammability, oxidative, toxic and chemical incompatibility properties of furfural showing some limitation pertaining to conventional hazardous-materials rating systems, and apprehend the need to develop adequate safety and emergency response procedures. In that regard, furfural is no exception.

## Conclusion

In conclusion, this review tries to summarize all catalytic processes for bio-based synthesis of furfural under the guiding criteria of the various kinds of catalysts that have been considered in reported scientific pertinent works. With respect to homogenous catalysis, mineral and organic acid catalysts mainly consisting of Brønsted acids are facing the need to get over a high activation barrier for furfural production. From this point of view, their combination with Lewis acid salts which benefit to D-xylose isomerization to D-xylulose, a more reactive intermediate, seems to be more promising option, since lower energy is required and higher furfural yield and selectivity could be obtained. However, the corrosive, environmental and handling problems should be taken into appropriate consideration with this kind of catalysts. As for ILs, even if extraordinary results are obtained, a major drawback pertains to their separation difficulty from the chemicals formed when used in biorefining, which still act today as a genuine barrier for their industrial applications. The limitations of solid catalysts basically lie in their complicated and therefore relatively costly synthesis processes, and easier tendancy to activation inhibition after reaction. Despite of these drawbacks, some of them do have inherent properties that might deserve valuable applications, such as sulfonated carbonaceous materials (SGO), zeolites (ZSM-5, SAPO-44, H-M) and partially hydroxylated MgF_2_ among others. The crucial application of a catalyst would be comprehensively evaluated by its efficiency, cost, chemical/thermal stability, reusability and so on. Additionally, the overall performance of a given catalyst may rely on adequate reactor engineering further improving catalytic furfural production as well as from smart furfural extraction downstream processing (e.g., making use of organic solvents, N_2_ stripping or permeation membrane) for consolidating economically-viable furfural production routes at industrial scale (meaning overpassing critical yields of furfural).

Keeping in mind the global hazardous-materials classification of furfural quoted in various regulatory frameworks, it becomes important to perform a complete process safety and end-use assessment that contributes to sustainable production and use the chemical in diverse industrial applications. These aspects must also be given prior importance like a number of furan-based compounds that have proven to entail adverse effects on the health and environment (e.g., dibenzofurans) whereas not produced on a voluntary basis. Clearly, regulations are set by pure conventions and physico-chemical properties of the chemical will persist irrespective of its official hazard classification. Though there are very limited safety concerns observed during the production of furfural from both homogeneous and heterogeneous catalytic point of view, care must be taken during the post-production phase in terms of classification, labeling and packaging of furfural and potential derivatives to avoid possible chemical accidents. Beyond conventions, risks associated with a chemical merely depend on the type of application and the surrounding environment. In this respect, examining furfural beyond its boundaries of conventional hazard classification would give a better understanding on the reactivity profile at various targeted applications, to avoid misleading conclusions and manage potential associated risks.

## Author contributions

FD, YW, AM, and KE analyzed the bibliography; FD, AM, GM, and CL wrote the paper.

### Conflict of interest statement

The authors declare that the research was conducted in the absence of any commercial or financial relationships that could be construed as a potential conflict of interest.

The author CL had previously collaborated with the reviewer AB.

## Footnotes

^1^CAMEO Chemicals. Chemical Datasheet n.d. https://m.cameochemicals.noaa.gov/chemical/3522 (Accessed September 27, 2016).

^2^Chemical Substances Bureau. EU Risk assessment - Furfural. 2008.
