# Correction: Hydrolysis of hemicellulose and derivatives—a review of recent advances in the production of furfural

**DOI:** 10.3389/fchem.2026.1827616

**Published:** 2026-03-20

**Authors:** Frontiers Editorial Office

**Keywords:** furfural, catalysis, green chemistry/safety biorefinery, biomass, dehydration, mechanism

In the published article, the **Conflict of Interest** statement lacked necessary details regarding a previous collaboration between the author Christophe Len and the reviewer Alina Mariana Balu. An investigation by Frontiers’ Research Integrity auditing team found that this conflict of interest did not unduly impact the peer review process or scientific validity of the article. The correct statement appears below:

“The authors declare that the research was conducted in the absence of any commercial or financial relationships that could be construed as a potential conflict of interest. The author CL had previously collaborated with the reviewer AB.”

The original article has been updated.

